# Primary production by the purple nonsulfur bacterium *Rhodopila globiformis* in an acidic, moderately sulfidic warm spring

**DOI:** 10.1128/aem.01217-25

**Published:** 2025-09-10

**Authors:** Kristopher M. Fecteau, Katelyn M. Weeks, R. Vincent Debes, Tanner J. Barnes, Kirtland J. Robinson, Joshua J. Nye, Melody R. Lindsay, Eric S. Boyd, Everett L. Shock

**Affiliations:** 1 School of Earth and Space Exploration, Arizona State University 7864, Tempe, Arizona, USA; 2 School of Molecular Sciences, Arizona State University 7864, Tempe, Arizona, USA; 3 Department of Microbiology and Cell Biology, Montana State University 33052, Bozeman, Montana, USA; Colorado School of Mines, Golden, Colorado, USA

**Keywords:** Yellowstone, carotenoid, acidophile, sulfide, anoxygenic photosynthesis, carbon fixation

## Abstract

Populations of the acidophilic purple nonsulfur bacterium *Rhodopila globiformis* were identified in two geographically distinct thermal areas in Yellowstone National Park (Wyoming, USA), as confirmed by 16S rRNA gene sequencing and detection of characteristic methoxylated ketocarotenoids. Microcosm-based carbon uptake assays where oxygenic photosynthesis was excluded via addition of 3-(3,4-dichlorophenyl)-1,1-dimethylurea yielded a light-driven dissolved inorganic carbon (DIC) assimilation rate (7 ± 2 mg C g C^−1^ h^−1^) comparable to those of highly productive algal mats in acidic hot springs, suggesting that *R. globiformis* may be performing photoautotrophy at the time of the assay. Rates of acetate assimilation were more than two orders of magnitude lower than DIC assimilation and did not differ between light and dark treatments, indicating photoheterotrophic use of acetate was not occurring, though photoheterotrophic assimilation of other organic compounds cannot be excluded. The tepid (35°C) spring waters are acidic (pH = 3.7) with moderate dissolved hydrogen sulfide (0.2 mM) and abundant DIC (11 mM), an apparently rare set of conditions thought to arise from extremely shallow mixing of oxygenated meteoric water and volcanic gases. Though originally isolated and cultured photoheterotrophically, in nature, *R. globiformis* may grow photoautotrophically under the normal conditions of its habitat, utilizing a stable supply of DIC afforded by the injection of CO_2_-rich volcanic gases. To our knowledge, these are the most acidic conditions under which light-driven DIC assimilation has been observed in the domain Bacteria.

**IMPORTANCE:**

Purple nonsulfur bacteria are ecologically diverse and metabolically versatile anoxygenic phototrophs; however, only a few acid-tolerant species are known. We identified populations of the purple nonsulfur bacterium *Rhodopila globiformis* in warm, acidic springs with moderate (~0.2 mM) concentrations of dissolved hydrogen sulfide in two thermal areas of Yellowstone National Park (Wyoming, USA). Comprehensive geochemical analyses of the spring waters illustrate that they are formed by mixing of groundwater and CO_2_-rich volcanic gases extremely close to the surface, relatively rare conditions that lead to characterization of *R. globiformis* as an endangered species. A high rate of light-driven assimilation of dissolved CO_2_ that rivals rates for acidophilic algae was observed, indicating that *R. globiformis* is responsible for a significant amount of primary production and suggesting it may primarily grow photoautotrophically in nature. These observations constitute the first insights into the physiological ecology of the most acidophilic anaerobic anoxygenic phototroph presently known.

## INTRODUCTION

Among anoxygenic phototrophic bacteria, purple nonsulfur bacteria in particular exhibit great metabolic flexibility, thought to grow best photoheterotrophically under anaerobic conditions but with most species also capable of photoautotrophy or chemoheterotrophic growth in the dark via either respiration or fermentation (1). Some species of purple nonsulfur bacteria may be considered extremophiles, including thermophilic, psychrophilic, alkaliphilic, and acidophilic members (1, 2). However, acidophilic anoxygenic phototrophs appear not to be taxonomically diverse (2, 3). A possible contributing factor to the apparent failure of anoxygenic phototrophs to more extensively radiate into acidic habitats is that extremely acidic habitats did not exist prior to oxygenation of the atmosphere following the evolution of oxygenic photosynthesis (4, 5), hundreds of millions of years after the diversification of anoxygenic phototrophs in anoxic, higher-pH environments. The apparent paucity of acidophily among anoxygenic phototrophic bacteria mirrors that of oxygenic phototrophic bacteria (i.e., *Cyanobacteria*), which are thought to be excluded from all environments with pH values below 4.0 (6), though the exact pH limit may be lower and difficult to discern (7). Thus, *Cyanobacteria* have apparently not been able to colonize many acidic environments made possible by their own metabolic production of oxygen. Instead, microbial phototrophy in acidic environments is dominated by algae (7–12). Nevertheless, a variety of acid-tolerant anoxygenic phototrophic bacteria have been characterized that grow aerobically rather than anaerobically, collectively referred to as the aerobic anoxygenic phototrophic bacteria (AAPB). Constituents of the AAPB are not capable of autotrophic growth and may be thought of as chemoheterotrophs that supplement energetic requirements via photophosphorylation (13, 14).

The diversity of anaerobic acidophilic anoxygenic phototrophs is presently limited to four species of purple nonsulfur bacteria across three genera within the *Alphaproteobacteria*. Strains of *Rhodoblastus acidophilus*, previously *Rhodopseudomonas acidophila* (15), were isolated from a variety of environments, including lakes, peat bogs, and swamps, and exhibited a pH minimum for growth of 4.8 and temperature optima of 25°C–30°C (16), though the lower pH limit is likely closer to 4.5 based on further study (17). Another species with similar characteristics, *Rhodoblastus sphagnicola*, was later isolated from a Sphagnum peat bog (18). *Rhodovastum atsumiense* was isolated from paddy soil; its temperature optima were found to be 30°C–35°C, and no growth was observed below a pH of 5.0 (19, 20). *Rhodopila globiformis*, originally *Rhodopseudomonas globiformis* (21), was isolated from a warm sulfur spring near the Gibbon River in Yellowstone National Park (YNP) in northwest Wyoming and was found to have a lower pH limit for growth of 4.2 and temperature optima of 30°C–35°C (22). Phylogenetically, its sister genus is *Acidiphilium*, a member of the AAPB found in a variety of acidic habitats, including acid mine drainage (23, 24) and geothermal environments such as YNP (7, 9, 12, 25), and it exists within the *Acetobacteraceae*, being closely related to the acetic acid bacteria (26).

**Fig 1:**
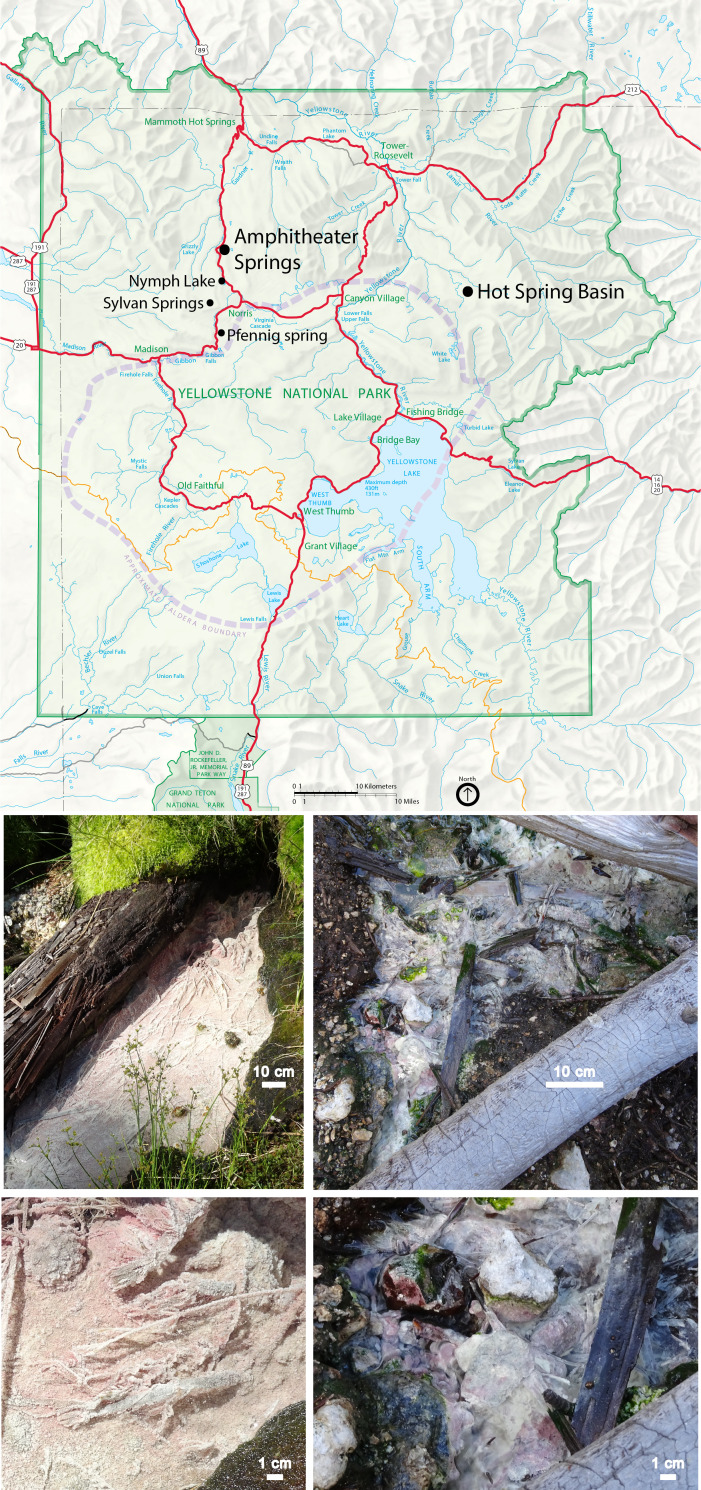
Top, map of Yellowstone National Park depicting sample locations from this study and other locations where *Rhodopila globiformis* has been observed (black points), major roads (red), the continental divide (orange), and the 0.64 Ma caldera boundary (purple), as well as topography and major park locations and water features. The map was derived from the nps.gov website, with selected layers removed in Adobe Illustrator. Bottom, photographs of the Amphitheater site (left) and the Hot Spring Basin site (right). Scale bars are 10 cm (top) and 1 cm (bottom). Photographs were taken during research conducted under research permit YELL-2017-SCI-5434.

*R. globiformis* has received significant attention for some unique biochemical characteristics, which contributed to it being reassigned to its own genus (21, 27). The species synthesizes both a high redox potential iron-sulfur protein (HiPIP) and a soluble cytochrome c_2_, each with particularly high redox potentials (28, 29). The cytochrome c_2_ was found to be similar to cytochromes associated with mitochondria and eukaryotic cytochromes based on its amino acid sequence (30) and crystal structure (31), respectively. The HiPIP has been extensively investigated electrochemically and spectroscopically (32–36), with observations suggesting it is involved in phototrophic electron transfer by reducing cytochromes associated with the reaction center (35). The main pigments of *R. globiformis* are bacteriochlorophyll *a* (22, 37) and methoxylated ketocarotenoids that have not been reported to occur in other species (38, 39). The light-harvesting 1-reaction center complex is membrane-bound via an N-terminal α-helix of the cytochrome subunit, rather than with acyl lipids as found in other purple bacteria, and includes polypeptides similar to the γ-polypeptides known only in species synthesizing bacteriochlorophyll *b* instead of bacteriochlorophyll *a* (40). *R. globiformis* produces menaquinone-9 and ubiquinone-9 (41, 42), as well as nearly equal abundances of rhodoquinone-9 and -10, a unique quinone composition when compared to members of the *Rhodospirillaceae* (43). The lipopolysaccharides were also found to be somewhat different from some other purple nonsulfur bacteria (*Rhodospirillium* spp.) in both the primary amino sugar (2,3-diaminoglucose) and hydroxyl fatty acid content (44). A novel strain of *Rhodopila*, likely a new species based on genetic differences, was isolated from a sulfidic spring in Lassen Volcanic National Park in northern California and found to produce 3-methylhopanoids, previously identified only in aerobic species (45). Elsewhere in YNP (Fig. 1), another strain of *R. globiformis* was isolated from a spring near Nymph Lake (2, 27), and *R. globiformis* was also detected via 16S rRNA gene sequencing with low relative abundances at two sample locations in the Sylvan Springs thermal area in the Gibbon Geyser Basin, ~3 km northwest of its original source spring (12). Nevertheless, in contrast to its biochemical attributes, there remains little known of its ecology.

Fundamental aspects of the physiology of *R. globiformis* examined in culture are inconsistent with what is thus far known of its natural habitat. The pH range for growth has been reported to be 4.2–6.5 (22, 27); however, the pH of the spring from which *R. globiformis* was first isolated was reported to be 3.0 (22) and other springs in the area also putatively harboring *R. globiformis* mostly have pH values 3–4, with some as low as 2.7 (27). The temperature of the source spring was reported to be approximately 40°C, which is its upper limit for growth in culture; in fact, no growth was observed at 40°C (22, 27). Additionally, sulfide was found to be growth inhibitory at low concentrations, yet the springs where *R. globiformis* has been found have been described as having large amounts of sulfur, suggesting the presence of sulfide (3, 27). A reduced sulfur source other than sulfide, such as thiosulfate or cysteine, was found to be required for growth (22), but concentrations of amenable sulfur sources are likely to be quite low in acidic springs. Thiosulfate concentrations were found to generally be less than 2 µM in acidic springs in YNP (46), and free amino acid concentrations have been observed at sub-micromolar levels in YNP hot springs (47). It was later determined that sulfate at concentrations below 1 mM could serve as an assimilatory sulfur source, with best growth at 0.1 mM, whereas growth-inhibitory dysregulation of the sulfate assimilation pathway was observed when sulfate exceeded 1 mM (48). Yet, since acidic spring waters in YNP are the result of the oxidation of sulfide to sulfuric acid (49–51), sulfate concentrations would be expected to approach or exceed 1 mM in springs with pH values of ~3. Finally, the organism was found to grow photoheterotrophically with a limited suite of organic compounds (including gluconate, mannitol, and fructose), a characteristic which could prove growth-limiting in nature, as there are likely to be only trace concentrations of specific organic compounds that are able to be assimilated by *R. globiformis*. However, its genome suggests the capability for metabolizing a more diverse array of organic substrates, suggesting there is a selective advantage for flexibility with respect to substrate usage (52).

Here, we report observations of two warm acidic springs in geographically distinct thermal areas in YNP not previously known to feature populations of *R. globiformis*. Physicochemical measurements were performed to characterize the habitat of *R. globiformis* and provide insights into the underlying hydrogeochemical processes resulting in the warm acidic waters of the springs. The microbial communities were investigated with 16S and 18S rRNA gene sequencing and complemented by analyses of phototrophic pigments, including identification of ketocarotenoids specific to *R. globiformis*. Microcosm-based carbon assimilation assays were conducted *in situ* to evaluate carbon metabolism in a natural population and related to inferences gleaned from stable isotope ratios of biomass and dissolved inorganic carbon (DIC). Collectively, these observations comprise the first evaluation of the physiological ecology of the most acidophilic anaerobic anoxygenic phototroph presently known.

## MATERIALS AND METHODS

### Field measurements and sampling

Temperature, pH, and specific conductance (conductivity normalized to 25°C) were measured using portable meters (YSI-30 and WTW 3110) as previously described (53). In 2017, dissolved oxygen was measured optically using a PreSens Fibox 4 meter and a DP-PSt3-L2.5-St10-YOP-HT sensor calibrated to 100°C (PreSens, Regensburg, Germany) as previously described (54); dissolved oxygen was determined colorimetrically in 2014 using the indigo carmine method with AccuVac Ampuls (Method 8316, Hach, Loveland, CO, USA) and a DR2400 spectrophotometer (Hach) according to the manufacturer’s protocol. Total dissolved sulfide was determined via the methylene blue method (Hach method 8131) using Hach reagents and DR2400 and DR1900 spectrophotometers in 2014 and 2017, respectively, on unfiltered water samples diluted with deionized water and analyzed immediately after collection. Total dissolved silica and ferrous iron were determined via the silicomolybdate (Hach method 8185) and the 1,10-phenanthroline (Hach method 8146) methods, respectively, using the same spectrophotometers on 0.2 micron-filtered (Supor membrane, Pall Corporation, Port Washington, NY, USA) water samples. Samples of microbial mat were collected aseptically in 2 mL cryovials and frozen immediately on dry ice and subsequently stored at −80°C until further processing. Reported elevations and Universal Transverse Mercator coordinates (zone 12T) are estimated from USGS 7.5 minute topographical maps and satellite imagery (Google Earth), respectively, as these were deemed more accurate than those obtained on site with a handheld GPS unit.

Filtered (0.2 micron, Supor membrane) water samples for laboratory analyses were collected and stored following previously described procedures (7). Samples for anions and cations were collected in 30 mL high-density polyethylene bottles that had been soaked and rinsed with multiple aliquots of deionized water; bottles for cations were spiked with 6 M methanesulfonic acid, resulting in a final concentration of ~20 mM, in order to prevent volatilization of ammonia or precipitation. These samples were frozen at −20°C as soon as possible after collection and maintained at that temperature until analysis. Samples for organic acid analysis were collected in 20 mL Qorpak amber glass bottles with PTFE cap inserts (55). The bottles were rinsed three times with deionized water, combusted (500°C, 24 hours), then sealed until sampling with the PTFE cap inserts that had been soaked in deionized water for 24 hours and dried. Samples were refrigerated at 4°C upon return to the laboratory until analysis. DIC samples were collected in acid-washed 40 mL amber glass vials and sealed with black butyl rubber septa without any headspace. Unfiltered DIC samples were also collected directly into vials at the sample site using a test tube holder. Dissolved organic carbon (DOC) samples were collected in combusted (450°C, 24 hours) 40 mL amber glass vials spiked with 0.1 mL of 85% phosphoric acid (Thermo Scientific, Waltham, MA, USA) and sealed with Teflon-lined septa without any headspace. Samples for stable isotope ratios of water were collected in 30 mL Qorpak square glass bottles that had been rinsed with deionized water and dried at 100°C (>12 hours); bottles were sealed without any headspace with gas-tight polymer-lined Polycone caps and stored at room temperature (~23°C).

### Geochemical analyses

Major anions (F^−^, Cl^−^, Br^−^, SO_4_^−2^, NO_3_^−^) and cations (Li^+^, Na^+^, K^+^, Mg^+2^, Ca^+2^, NH_4_^+^) were determined on separate Dionex DX-600 4 mm ion chromatography systems using suppressed-conductivity detection as previously described (9). Total (carboxylic acid + carboxylate) acetate and formate concentrations were analyzed with the same Dionex DX-600 system used for major anions but equipped with AG-/AS-24A anion exchange columns. Injections of undiluted samples were from an AS40 autosampler using 0.5 mL vials (one injection/vial) onto a 100 µL sample loop. The eluent was held isocratically at 2.5 mM for 20 minutes, after which the eluent concentration was raised to 45 mM over 5 minutes then held at 45 mM for 9 minutes to elute other ions, and finally, the eluent was returned to 2.5 mM over 1 minute and re-equilibrated at 2.5 mM hydroxide for 10 minutes prior to the next injection. The flow rate was constant at 1 mL/minute, and the suppressor current was 112 mA. A linear calibration curve was constructed from a series of solutions prepared using 1,000 µg/mL acetate and formate standards (High Purity Chemicals, Charleston, SC, USA) over the concentration range of 2–750 µg/L (acetate) or 8–750 µg/L (formate). The solutions also contained 2 mg/L fluoride obtained from a 1,000 µg/mL mixed anion standard (Environmental Express) in an effort to match the standard and sample matrices, as acetate elutes on the tail of the fluoride peak.

Analyses of DIC and DOC were performed with an OI Wet Oxidation TOC analyzer coupled to a Thermo Delta Plus Advantage IRMS as described previously (9). Isotope ratios were standardized via analysis of solutions of three glycine working standards: low, δ^13^C = −39.64‰, δ^15^*N* = 1.35‰; mid, δ^13^C = −8.36‰, δ^15^*N* = 27.9‰; high, δ^13^C = 15.67‰, δ^15^*N* = 51.8‰, versus Vienna Pee Dee Belemnite (VPDB) and air, respectively. Stable isotope ratios of water were measured via cavity ring-down spectroscopy using a Los Gatos Research DLT-100 isotope analyzer as previously described (56).

Charge imbalances were calculated using the geochemical speciation code EQ3/6 (57) using activity coefficients calculated via an extended Debye-Hückel equation and equilibrium constants derived from the Helgeson-Kirkham-Flowers equation of state (58, 59). Calculations were performed via the AqEquil Python package (60) accessed from the Water-Organic-Rock-Microbe portal (worm-portal.asu.edu). Charge imbalance is reported as the percent of the mean charge (49).

### DNA extraction, PCR, and 16S/18S rRNA gene sequencing

DNA was extracted from thawed mat samples (~0.25 g; excluding Lemonade Creek) using the ZymoBIOMICS DNA miniprep kit with a stated binding capacity of 25 µg according to the manufacturer’s instructions. Excess sample fluid was removed via centrifugation prior to the addition of lysis buffer and bead beating. Extracted DNA was screened for bacterial 16S rRNA genes by polymerase chain reaction (PCR) using 1100F/1492R primers (annealing temperature of 55°C) as previously described (61). Additionally, the same PCR methods were used to screen for the presence of *bchY* to generate ~500 base pair amplicons using previously described primers and an annealing temperature of 50°C (62).

DNA extracts were sent to the Arizona State University (ASU) Genomics Facility for next-generation sequencing of 16S and 18S rRNA genes using the Earth Microbiome Project primer sets (515F/806R and 1391F/EukB, respectively) and protocol (63–65). Only the DNA extracts from samples collected in 2017 are described herein, with 18S rRNA gene sequencing being performed only for the Amphitheater site. Sequencing was performed on an Illumina-MiSeq (v2, 2 × 250). The FastQ files received from the ASU Genomics Facility underwent quality analysis using FastQC (66). The “.fasta” files were uploaded to QIIME2 (v. 2023.7) for sequence cleaning, trimming, and amplicon sequence variant (ASV) generation using DADA2 (67, 68). The SILVA genes database (v. 138.1) was used for taxonomic classification via the RESCRIPt plug-in available on QIIME2 (69). ASVs that remained inadequately assigned after taxonomic classification via QIIME2 were manually classified using BLASTn via the National Center for Biotechnology Information (NCBI) website (https://blast.ncbi.nlm.nih.gov). The conversion of the sequence counts to percent relative abundances was performed in R (70) using the vegan package (v 2.6-4; 71). Plotting of the percent relative abundances was performed using Origin software (v. 2023 b.).

### Pigment analysis

Acetone-soluble pigments were extracted from thawed mat samples collected at the Amphitheater spring in 2017 using bead beating in multiple aliquots of 7:2 (vol:vol) acetone:methanol as previously described (7). The pooled extract was analyzed using liquid chromatography-diode array detection-tandem mass spectrometry (LC-DAD-MS/MS) on an Agilent 1290 high-performance liquid chromatography (HPLC) system coupled to an Agilent 6530 accurate mass quadrupole-time of flight mass spectrometer (Agilent Technologies, Santa Clara, CA, USA). Fifty microliters of sample were injected onto a YMC Carotenoid C30 reverse-phase column (3 × 250 mm × 5 µm particle size; YMC, Kyoto, Japan) and eluted at a constant flow rate of 0.425 mL/minute using a solvent system modified after that of Sander et al. (72). The eluent was initially isocratic with 100% solvent A (81:15:4 methanol:methyl *tert*-butyl ether:water) for 30 minutes followed by a linear gradient to 100% solvent B (6:90:4 methanol:methyl *tert*-butyl ether:water) at 90 minutes. At that time, the eluent was returned to 100% A over 1 minute and then re-equilibrated for 19 minutes. The eluent system was adapted for use with an HPLC system equipped with a binary pump by preparing the eluent endmember compositions to construct the gradient; the solvent B mixture was biphasic, with the top, organic-rich phase being used for chromatography. Pigments were monitored via the diode array detector (Agilent 1260) at 360, 475, and 665 nm and spectra were collected from 325–800 nm at 10 Hz. The eluate was then directed into the mass spectrometer and ionized using atmospheric pressure chemical ionization in positive ionization mode with parameters based on those of van Breemen et al. (73). Specific ionization conditions were a corona current of 8 µA, vaporizer temperature of 350°C, ionization gas flow rate of 10 L/minute with a temperature of 325°C, and nebulizer gas pressure of 45 psi. Mass spectra were collected from 200 to 1,500 m/z at a scan rate of 1 Hz. Data-dependent MS/MS spectra were obtained for the most abundant ion in each cycle (if >0.01% relative abundance and >200 counts) at fixed collision energies of 15, 30, or 45 V with argon as the collision gas. Mass accuracy was verified prior to analysis, and accurate mass (<2 ppm) was maintained by infusion of an internal reference mass solution for mass correction over the course of the chromatographic analysis.

### Carbon assimilation assays and solid-phase carbon/nitrogen analyses

Rates of biological DIC assimilation and acetate assimilation and oxidation to DIC (i.e., dissimilation or mineralization) were evaluated using microcosms spiked with ^14^C-enriched substrates with methods modified from those previously reported (10, 74). In the laboratory prior to field work, 20 mL serum bottles were crimp-sealed with butyl rubber septa and flushed extensively with ultra-high purity nitrogen. In the field, the bottles were equilibrated to the lower local atmospheric pressure by removal of excess nitrogen via a plastic syringe and 16-gauge needle. Spring water was collected using a 160 mL syringe and aliquoted to a 10 mL syringe via a 3-way stopcock to minimize gas exchange prior to injection of 10 mL of spring water into each bottle. Benthic biomass/sulfur and spring water were collected aseptically in a 50 mL Falcon tube, and the tube was shaken vigorously to yield a slurry. To prepare microcosms, 1 mL of slurry was injected via a 1 mL plastic syringe and 16-gauge needle into each bottle. A set of microcosms designated as killed controls was spiked with 0.1 mL of 50 mM HgCl_2_. A separate set of microcosms was wrapped in aluminum foil to evaluate light-independent rates. In 2017, all microcosms for the DIC assay were spiked with 0.1 mL of 1 mM 3-(3,4-dichlorophenyl)-1,1-dimethylurea (DCMU) in 50% ethanol (vol/vol) to exclude photosystem II-dependent (i.e., oxygenic) photosynthesis; in 2014, DCMU was not employed, nor was it employed in acetate assays. All assays (light, dark, and killed) were conducted in triplicate. To initiate the assays, microcosms were injected with 0.2 mL solution of either NaH^14^CO_3_ (54 mCi/mmol) or Na^14^CH_3_^14^COO (110 mCi/mmol), resulting in final activities of 5.3 µCi and 0.71 µCi per vial, respectively. Microcosms were incubated in the source of the spring for 60 minutes, then immediately placed in the dark on dry ice to terminate the assay and subsequently stored at −20°C until further processing.

Aliquots of remaining slurry (1 mL; *n* = 6) were collected in 2 mL cryovials, frozen on dry ice in the field, and stored at −20°C until processing. Thawed aliquots were individually collected on pre-weighed plastic weighing boats, dried at ~80°C for 3 days, and then weighed to determine the dry mass added to each microcosm. The dried biomass was then pooled, homogenized with an agate mortar and pestle, and milligram quantities were aliquoted into tin capsules using a microbalance. Sealed capsules were analyzed for C and N contents and stable isotope ratios using an ECS 4010 Elemental Analyzer (Costech Analytical Technologies, Valencia, CA, USA) coupled to a Thermo Delta Plus Advantage isotope ratio mass spectrometer (IRMS). The resulting CO_2_ and N_2_ were separated via gas chromatography, and their molecular ions were monitored as ion chromatograms at 44 m/z and 28 m/z, respectively, with peak areas related to elemental abundances via linear calibration curves using NIST 2710 (Montana soil). Carbon isotope ratios were standardized using the same glycine working standards employed for DIC and DOC analyses. Carbon and nitrogen analysis for the Hot Spring Basin site was also completed using the same methods with a thawed mat sample aliquot.

For analysis, microcosms were allowed to thaw and then injected with 1 mL of 1 M HCl to volatilize DIC. Vials for DIC assimilation assays were unsealed and allowed to equilibrate in a fume hood dedicated to radioisotope work for >2 hours, whereas acetate assay vials remained sealed during equilibration (also >2 hours). To evaluate rates of acetate dissimilation (oxidation of acetate to DIC), 5 mL of headspace in each acetate assay vial was removed via a 10 mL syringe and stopcock and then injected into an evacuated 12 mL serum bottle containing 1 mL of Carbo-Sorb E (Perkin-Elmer, Santa Clara, CA, USA) and allowed to equilibrate at 4°C for 16–24 hours. The Carbo-Sorb E solution was then transferred to scintillation vials for liquid scintillation counting (described below). To estimate the fraction of the headspace removed for this assay, the total volume of sealed 20 mL bottles was estimated by comparing the mass of bottles filled with deionized water and sealed without headspace versus the initial combined mass of each individual empty bottle, cap, and septum and using the density of pure water at the laboratory temperature to calculate the associated volume. The total volume of the headspace after expansion into the syringe was then calculated by subtraction of the known volumes of liquids/solids added to each assay vial. The calculated fraction of the 5 mL sample (0.25) was subsequently employed in the calculation of the dissimilation rates (see below).

After equilibration, the contents of the microcosm vials were filtered onto 0.2 µm pore size, 25 mm diameter Nucleopore polycarbonate membranes (Whatman, Florham Park, NJ, USA). The filters were washed with ~10 mL of deionized water and dried in scintillation vials at ~80°C for ~16 hours. Ten milliliters of Cytoscint ES scintillation cocktail (MP Biomedicals, Irvine, CA, USA) were added to each scintillation vial (both those containing dried filters and those containing Carbo-Sorb E), ensuring that filters were completely submerged in the fluid. Radioactivity associated with each vial was measured using a LS 6500 liquid scintillation counter (Beckman Coulter, Indianapolis, IN, USA), with disintegrations per minute (DPM) being calculated from counts per minute via a quench curve. Substrate conversion rates were then calculated as


(1) $$\begin{array}{c} mg\;C\;gC^{-1}h^{-1}=\;\frac{\left({DPM}_{T}-{DPM}_{K}\right)\;\left({mmol}_{C}\right)\;\left(M_{C}\right)\;\left(\nu_{C}\right)\;\left(\alpha \right)}{\left({DPM}_{added}\right)\;\left(gdw\right)\;\left(\frac{wt\;\%\;C}{100\%}\right)\;\left(t\right)\;\left(F_{s}\right)},\; \end{array}$$

where DPM_*T*_ is the mean measured DPM in each treatment, DPM_*K*_ is the mean DPM in the associated killed control, mmol_*C*_ is the total (labeled plus native) amount of substrate in each microcosm, *M*_*C*_ is the molar mass of carbon, *ν*_*c*_ is the number of moles of carbon per mole of substrate, *α* is the isotopic discrimination factor (1.05; 75), DPM_added_ is the amount of radioactivity added to each microcosm, gdw is the dry mass of mat material in each microcosm in grams, *t* is the duration of the assay in hours, and *F*_*s*_ is the fraction sampled for measurement (1 for assimilation rates, 0.25 for dissimilation rates, see above). Uncertainties in each variable above were propagated through the calculation and reflected in the reported uncertainties in the rate, though the largest source of uncertainty is the experimental variation of the microcosm replicates. To summarize, the rate is the fraction of radioactive substrate converted, scaled to the total amount of substrate in each assay, normalized to experimental duration and solid-phase carbon (as a proxy for biomass amount). While substrate transformation in the microcosms presumably follows first-order kinetics (i.e., the “bottle effect”), the rates are approximated using zero-order kinetics given the extremely small extents of substrate conversion over the short duration of the experiment.

## RESULTS AND DISCUSSION

### Field sites

Two acidic, warm springs with flocculent pink and yellow mats, thought to represent benthic suspensions of *Rhodopila globiformis* cells associated with precipitated sulfur, were located in geographically distinct thermal areas in YNP (Fig. 1). The primary study site is located in Amphitheater Springs, a thermal area along the Norris-Mammoth corridor outside of the 0.64 Ma caldera ~5 km north of Nymph Lake, where other springs hosting *R. globiformis* have been reported, and ~13 km north from where it was originally isolated (2, 27). The spring is located in the extreme southern portion of the thermal tract along Lemonade Creek, the primary drainage of the basin, and has a distinct source physically isolated from the creek, into which its effluent flows. There are also locations nearby this spring in the creek bottom where bubbling gas and similar pink and yellow mats are evident. Upstream of the spring, the physicochemical data provided herein (Table 1) indicate Lemonade Creek has received minimal hydrothermal influence, whereas downstream in the extreme northern portion of the basin, its waters consist primarily of the hot (>60°C) effluents from several acidic, sulfur-depositing, high-discharge springs located along and near the creek (76). Downstream of this input, once the creek waters have cooled to below the upper temperature limit for photosynthesis under acidic conditions (~56°C; 77), extensive mats of the acidophilic alga *Cyanidioschyzon merolae* cover the creek bed (78–82). The second site is located in Hot Spring Basin, an extensive, remote, gas-rich thermal area in the Mirror Plateau region, also outside of the 0.64 Ma caldera (83–86).

**TABLE 1:** Physicochemical data

	Amphitheater 2017	Amphitheater 2014	Lemonade Creek	Hot Spring Basin
Sample ID (YYMMDD)	170722TO	140803SK	170722TP	170730TQ
Easting	0521755	0521755	0521760	0557919
Northing	4960245	4960245	4960228	4954198
Elevation, m	2,289	2,289	2,295	2,563
Temperature, °C	35.4	38.0	12.6	37.2
Specific conductance, µS/cm	308.0	422.6	50.1	1,021
pH (field/calculated)	3.76/3.77	3.52/3.55	5.28/5.47	2.95/2.39
Sulfide, µM	210	120	0.65	90
Dissolved oxygen, µM	2.0	8.9	180	16
Silica, mM	2.6	2.5	1.0	2.9
Fluoride, mM	0.0031	0.0065	0.0026	0.022
Chloride, mM	0.079	0.10	0.016	0.021
Bromide, µM	0.13	0.12	0.032	0.060
Sulfate, mM	1.0	1.3	0.058	4.2
Nitrate, µM	0.13	0.93	0.15	0.62
Lithium, µM	3.1	4.0	0.17	3.0
Sodium, mM	0.97	1.2	0.082	1.1
Potassium, mM	0.43	0.53	0.045	0.58
Magnesium, mM	0.056	0.075	0.034	0.11
Calcium, mM	0.13	0.18	0.073	0.23
Ammonium, µM	32	34	0.41	280
Ferrous iron, µM	57	53	0.36	50
Formate, µM	2.5	Not measured	Not measured	2.3
Acetate, µM	0.83	Not measured	Not measured	0.99
δ^2^H-H_2_O, ‰ vs VSMOW	−136.3 ± 0.6	−140.1 ± 0.3	−133.0 ± 0.6	−136.9 ± 0.7
δ^18^O-H_2_O, ‰ vs VSMOW	−18.0 ± 0.2	−18.0 ± 0.1	−17.7 ± 0.2	−17.3 ± 0.1
DIC, mM	11	7.9	2.1	6.0
δ^13^C-DIC, ‰ vs VPDB	−3.5 ± 0.2	−4.3 ± 0.2	−6.4 ± 0.4	−3.5 ± 0.2
DOC, mM	0.12	0.13	0.16	0.088
δ^13^C-DOC, ‰ vs VPDB	−25.4 ± 0.2	−24.5 ± 0.3	−26.7 ± 0.2	−25.4 ± 0.6
Mat C, wt % (dry mass basis)	9.11 ± 0.07	10.63 ± 0.07	Not measured	12.0 ± 0.3
δ^13^C-Mat, ‰ vs VPDB	−26.4 ± 0.1	−26.1 ± 0.1	Not measured	−25.6 ± 0.1
Mat N, wt % (dry mass basis)	1.315 ± 0.007	1.355 ± 0.005	Not measured	0.58 ± 0.01
δ^15^N-Mat, ‰ vs air	−2.8 ± 0.1	−4.9 ± 0.1	Not measured	−5.9 ± 0.1
Sediment C:N, mol:mol	8.08 ± 0.07	9.15 ± 0.07	Not measured	24.1 ± 0.7
Δ^13^C, ‰	−22.9 ± 0.2	−21.8 ± 0.2	Not measured	−22.1 ± 0.2
Charge imbalance, %	0.30	1.1	23	−69

### Community composition

**Fig 2:**
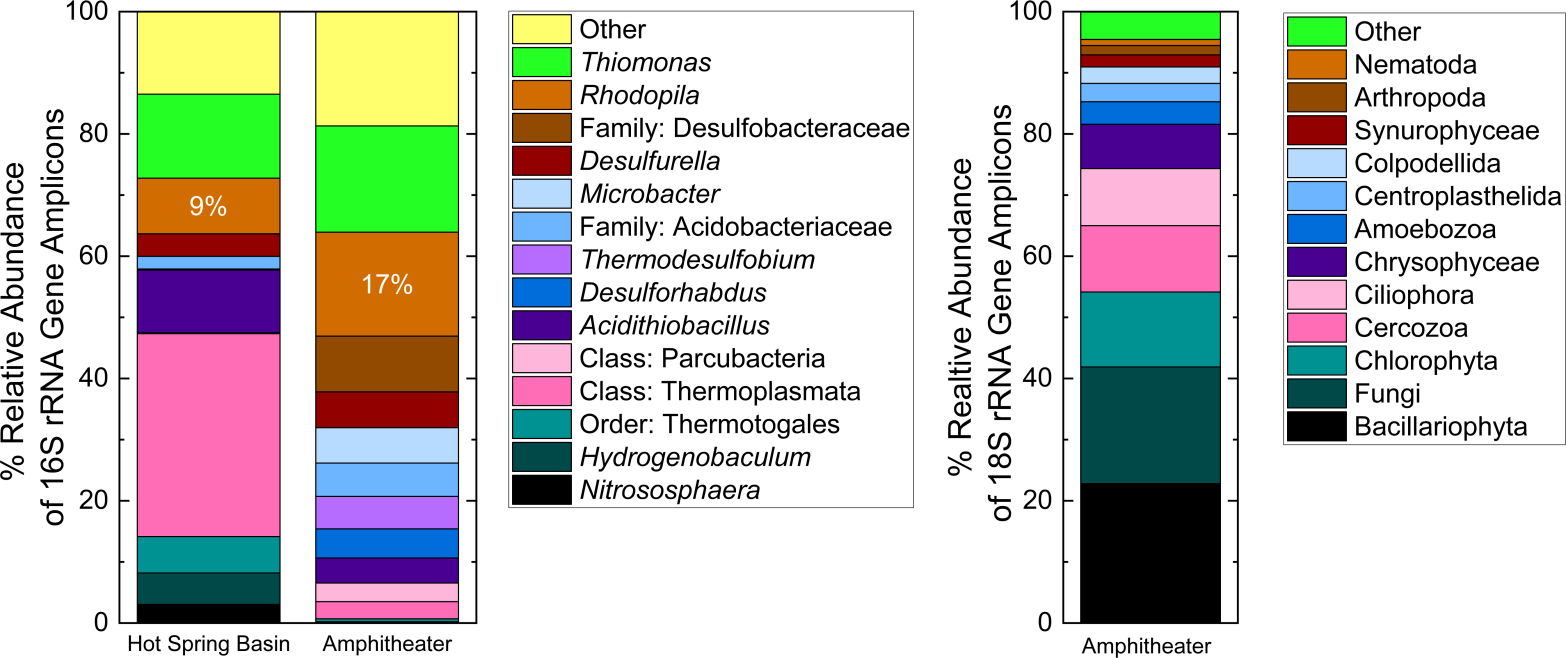
Taxonomic affiliations of 16S (left) and 18S (right) rRNA gene sequences, with those for *Rhodopila* explicitly indicated. Sequence results correspond to DNA extracted from samples collected in 2017; 18S rRNA gene sequencing was completed for the Amphitheater site only.

Amplicons for *bchY*, the gene encoding the Y subunit of chlorophyllide oxidoreductase, the first enzyme in the chlorophyll biosynthetic pathway specific for bacteriochlorophylls, and therefore, a genetic marker for anoxygenic phototrophs (62), were obtained from DNA extracted from both sample sites, confirming the presence of anoxygenic phototrophic bacteria. As hypothesized, 16S rRNA gene ASV affiliations indicated that *Rhodopila* was one of the most abundant genera, and the most abundant bacterial phototroph, in both the Amphitheater and Hot Spring Basin spring communities (17% and 9%, respectively) (Fig. 2). The most abundant ASV at both sites exhibited 100% sequence similarity to the type strain, *R. globiformis* DSM 161^T^. Only the genus *Thiomonas* had a higher relative abundance at the Amphitheater site (Fig. 2), characterized as being facultatively chemolithoautotrophic with the ability to use sulfur compounds such as elemental sulfur, sulfide, or thiosulfate as electron donors (87, 88). Strains of *Thiomonas* have been implicated in the oxidation of arsenite in arsenic-rich acidic hot springs in YNP (89, 90), yet the total dissolved arsenic concentration at the Amphitheater site is several orders of magnitude lower (0.16 µM [E. L. Shock, unpublished data]). In the Hot Spring Basin spring, *Acidithiobacillus,* as well as *Thiomonas*, both possibly active in sulfur and/or sulfide oxidation, were more abundant than *Rhodopila*, whereas the most abundant taxon was the archaeal class *Thermoplasmata*, though the most abundant ASV exhibited poor sequence similarity (86%) with cultured representatives. Other notable taxa assigned to significant ASVs at the Hot Spring Basin site are the hydrogen oxidizer *Hydrogenobaculum* (99% sequence similarity [91]) and the archaeal ammonia oxidizer *Nitrososphaera* (83%–85% sequence similarity [92]). Most other abundant taxa in the springs are known to be involved in sulfur cycling, including *Desulfobacteraceae*, *Desulfurella*, *Thermodesulfobium*, and *Desulforhabdus* (Fig. 2).

16S rRNA gene sequences most closely affiliated with other bacterial phototrophs exhibited significantly lower relative abundances (<1%) than those affiliated with *Rhodopila* in the Amphitheater and Hot Spring Basin spring communities, including those affiliated with *Rhodoblastus*, *Rhodovastum*, and *Rhodobacter*, as well as the AAPB genera *Acidiphilium* and *Acidisphaera*. No sequences were most closely affiliated with *Cyanobacteria*. 18S rRNA gene sequences obtained from the Amphitheater spring include several taxa of algae as major constituents, specifically *Bacillariophyta* (diatoms), *Chlorophyta* (green algae), *Chrysophyceae*, and *Synurophyceae* (Fig. 2). In contrast, *Rhodophyta* comprised <1% of the 18S rRNA gene ASVs, and these were not most closely affiliated with *Cyanidiophyceae*, a class of unicellular rhodophytes that includes *C. merolae*, known to dominate phototrophic mats in acidic aquatic environments in YNP above 40°C, e.g., downstream portions of Lemonade Creek (8, 78, 79, 81). Fungi collectively comprised the largest non-phototrophic eukaryotic taxon (19% relative abundance), exceeded only by *Bacillariophyta* (23% relative abundance).

### Pigment composition of the Amphitheater mat

**Fig 3:**
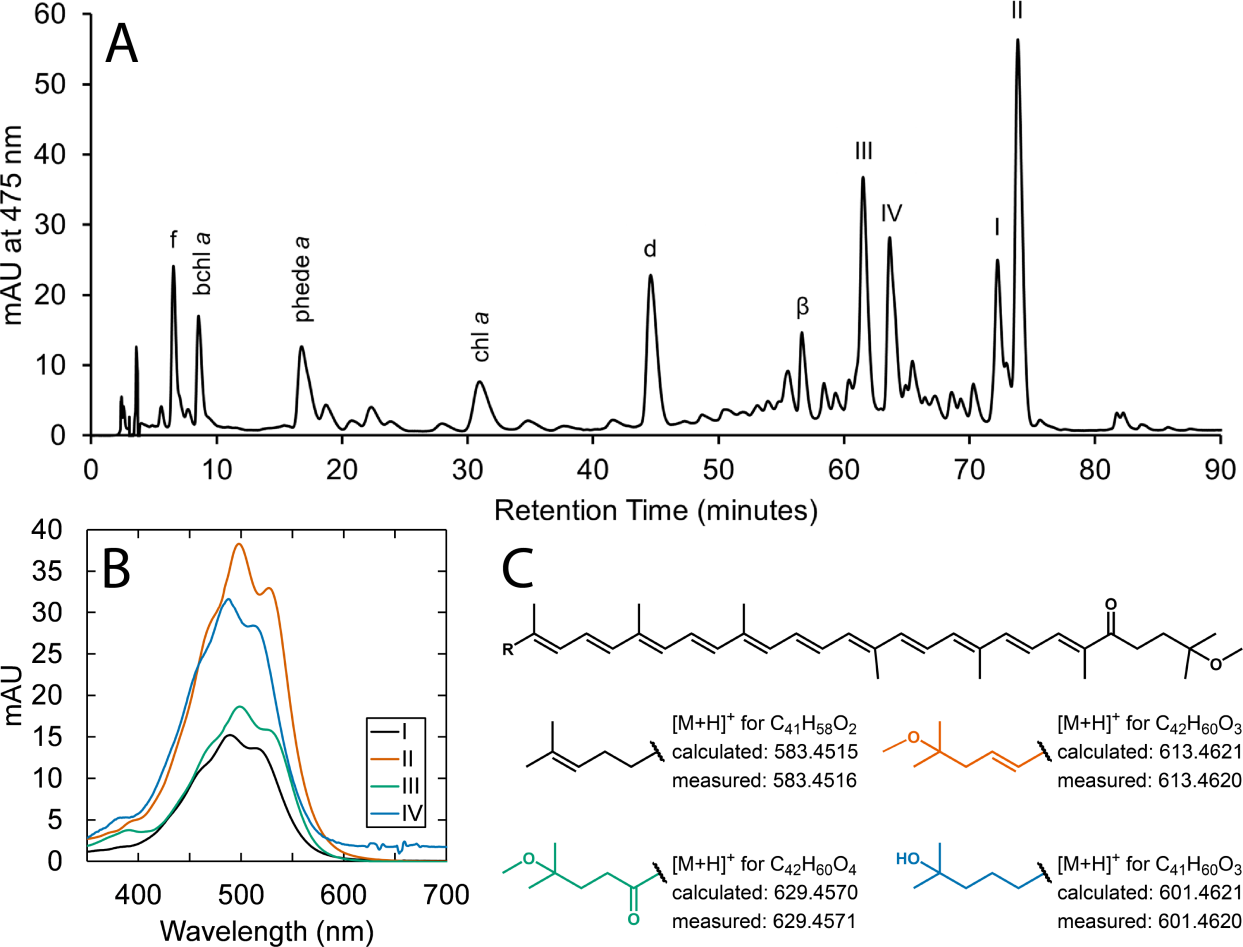
(**A**) Diode array chromatogram at 475 nm of the pigment extract of Amphitheater sediments. The four ketocarotenoids diagnostic for *Rhodopila globiformis* are indicated with Roman numerals; other major peaks are labeled with their assignments as follows: f, fucoxanthin; bchl *a*, bacteriochlorophyll *a*; phede *a*, pheophorbide *a*; chl *a*, chlorophyll *a*; d, diatoxanthin; and β, β-carotene. (**B**) Diode array spectra of the four ketocarotenoids. (**C**) General structure of the ketocarotenoids (top) with the variable terminal R-groups colored as in (**B**) and high resolution mass to charge ratios for the molecular ions in the corresponding mass spectra averaged over the retention time of the diode array response (bottom). Further details are compiled in the supplemental material (Table S1).

*R. globiformis* synthesizes several unique methoxylated ketocarotenoids that have not been identified in other species (38–40). Analysis of pigment extracts from mat material collected at the Amphitheater site by LC-DAD-MS/MS confirmed the presence of the four ketocarotenoids (R.g. keto I–IV) previously identified under normal culture conditions (Fig. 3), supporting the sequencing results in demonstrating *R. globiformis* as a predominant phototroph in the mat. The online diode array spectra yielded absorption maxima in agreement with those previously reported (38), and the high-resolution molecular ions were consistent with the molecular formulas of the carotenoids. Collision-induced fragmentation of the molecular ions yielded fragments resulting from loss of methanol due to cleavage of one (M+H−32) or both (M+H−64) methoxy groups (or, in the case of IV, M+H−50, due to loss of a methoxy group and a hydroxyl group), consistent with the carotenoid structures.

A predominant chlorophyll in the pigment extract was bacteriochlorophyll *a*, which is the primary chlorophyll employed by *R. globiformis* (22, 37). Chlorophyll *a* was also detected, indicating the presence of oxygenic phototrophs and consistent with the presence of algae indicated by 18S rRNA gene sequencing. Degradation products of these chlorophylls, including bacteriopheophytin *a*, pheophytin *a*, pheophorbide *a*, and pyropheophorbide *a*, were also identified, all of which are common in moderately acidic springs (7). Aside from the ketocarotenoids associated with *R. globiformis*, other abundant carotenoids in the pigment extract were identified as diatoxanthin and fucoxanthin, carotenoids found in diatoms and other algae, in particular other Heterokont taxa, including *Chrysophyceae* and *Synurophyceae* (39, 93, 94), consistent with the 18S rRNA gene sequencing results. There was, however, no evidence for the presence of chlorophylls *b* or *c*, inconsistent with expectations given the algal taxa detected via 18S rRNA gene sequencing, except that pheophytin *c*_1_ and pheophytin *c*_2_ were identified, which possibly are the result of pheophytinization (i.e., loss of magnesium) of chlorophyll *c*. There were also no mass spectra consistent with Zn-bacteriochlorophyll *a*, which is synthesized by members of the genus *Acidiphilium* (95, 96) and has been detected in some other moderately acidic YNP springs (7). In spite of the enhanced resistance to pheophytinization of Zn-bacteriochlorophyll *a* (97), thought to be advantageous for acid tolerance by *Acidiphilium*, its sister genus *Rhodopila* apparently does not chelate bacteriochlorophyll *a* with zinc to a detectable extent, certainly not to the extent observed in *Acidiphilium*. A compilation of the results of the pigment analysis is located in the Supplemental material (Table S1).

### Rates of inorganic and organic carbon assimilation

**Fig 4:**
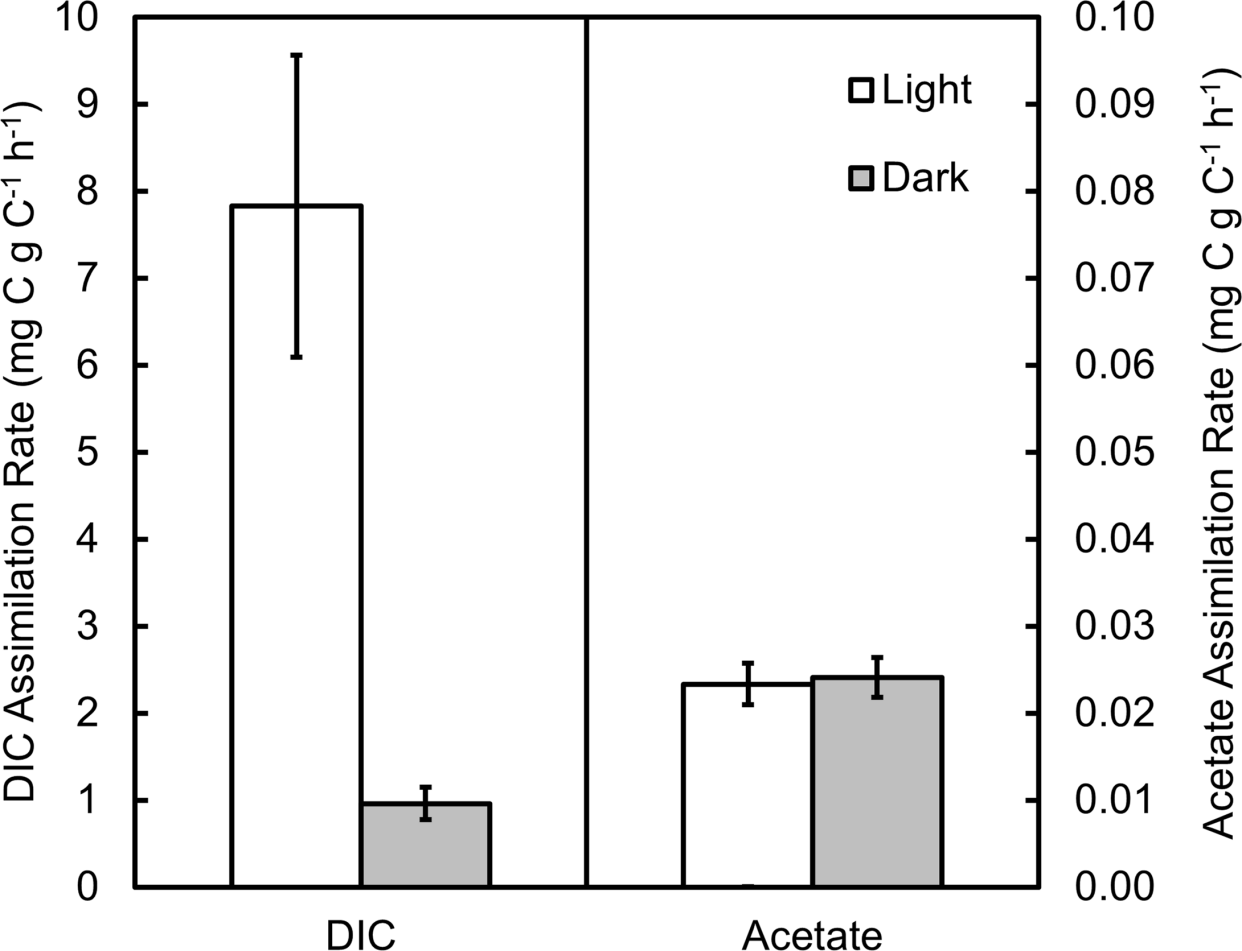
Rates of DIC assimilation (left) and acetate assimilation (right) in microcosms containing mat and spring water from the Amphitheater site in 2017. Microcosms were incubated *in situ* and were either exposed to light (white bars) or were excluded from light using aluminum foil (gray bars). Microcosms for assessing DIC assimilation were amended with 3-(3,4-dichlorophenyl)-1,1-dimethylurea to exclude oxygenic photosynthesis.

Microcosm carbon assimilation experiments were conducted at the Amphitheater site to assess the carbon metabolism of the microbial community. In 2017, a significantly higher rate of DIC assimilation was observed in microcosms exposed to light than in those excluded from light, indicating light-driven DIC assimilation was occurring in the mat at the time of the assay (Fig. 4). These microcosms were amended with DCMU to a final concentration of 10 µM, which is effective at decreasing light-driven assimilation of DIC to levels close to those observed in the dark in other acidic microcosms (Fig. S1), such as microcosms from the outflow of Dragon Spring where the predominant phototrophs are algae (10, 98). Thus, assimilation by algae in microcosms amended with DCMU is negligible, and the light-driven DIC assimilation rate observed at the Amphitheater spring may be attributed to anoxygenic phototrophs such as *R. globiformis*.

**Fig 5:**
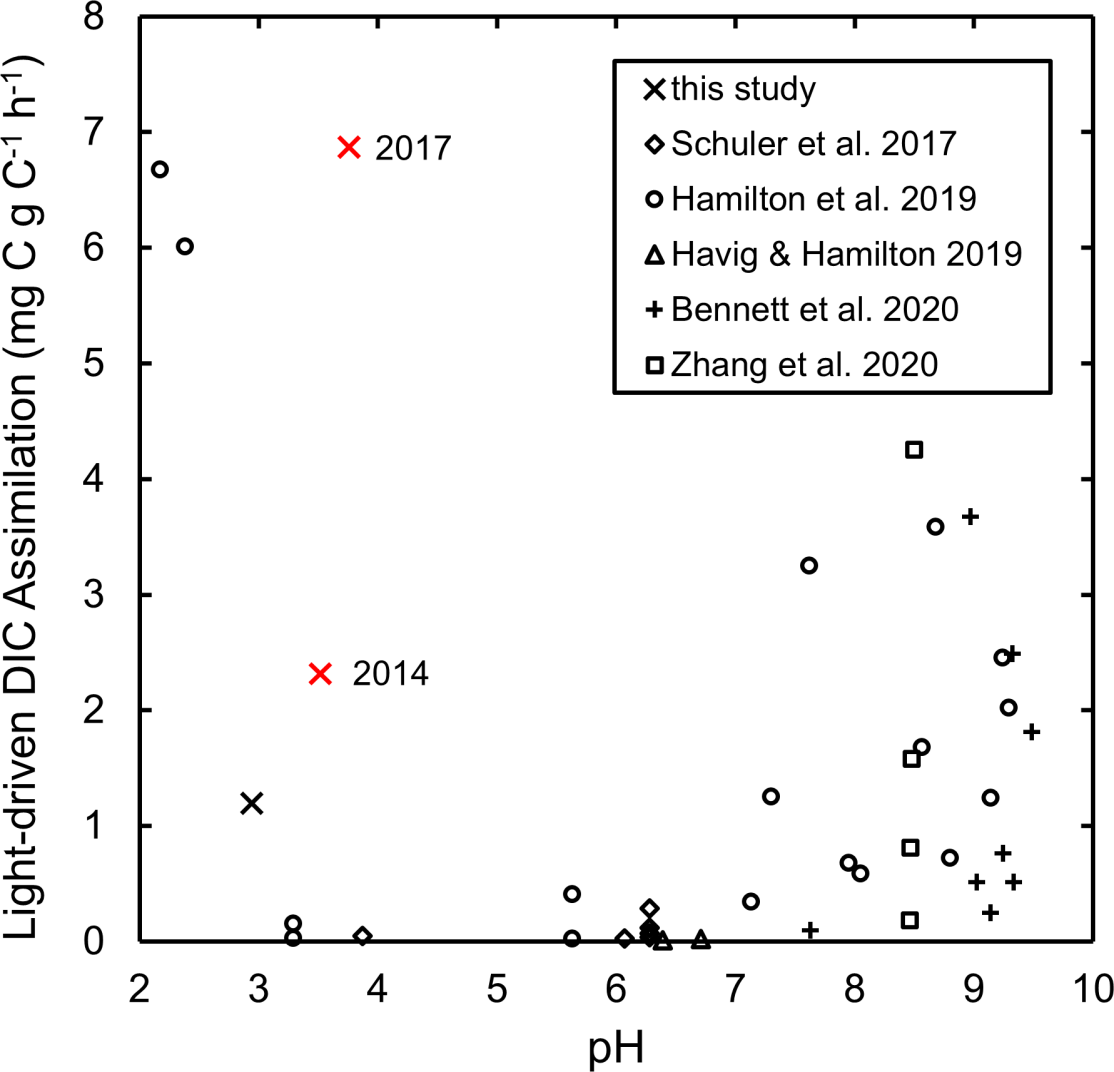
Rates of light-driven DIC assimilation at the Amphitheater site in 2014 and 2017 (red crosses; Table S3) and rates at other hot spring locations in Yellowstone and Tengchong, China (black symbols) reported in the literature (12, 104–107) or included in this study (Table S4) versus the pH of the hot spring fluids at the sample location. Rates are calculated as the difference between rates measured using microcosms exposed to light and those determined using microcosms excluded from light (wrapped in aluminum foil). Rates for spring locations in China were calculated as the average rate over the afternoon (1–6 PM) photoperiod (107). The year that the rate was determined is indicated for the Amphitheater site. The rate at the outflow of Dragon Spring (Table S4) is not included, as only a minimum rate could be determined. For clarity, uncertainties in the rates are not depicted; rates that were not statistically distinguishable from zero are not included in the plot.

The light-driven DIC assimilation rate in 2017 was 7 ± 2 mg C g C^−1^ h^−1^, which is similar to, or in some cases greatly exceeds, rates of light-driven DIC assimilation measured in the absence of DCMU in some other acidic hot springs in YNP where algae (*C. merolae*) are the major phototrophs (Fig. 5) (12). As is the case for most purple nonsulfur bacteria (1), the genome of *R. globiformis* contains all genes necessary for carbon fixation via the reductive pentose phosphate cycle (i.e., Calvin cycle [52]). Such a high rate of light-driven DIC assimilation, rivaling or exceeding that of algae, suggests *R. globiformis* may be growing photoautotrophically under the conditions at the time of the assay. However, purple nonsulfur bacteria are known to employ the Calvin cycle as a means of dissipating excess reducing power to maintain redox balance during photoheterotrophic growth (99–103). For example, in wild-type *Rhodopseudomonas palustris,* it was estimated through ^13^C-metabolic flux analysis that 15.9% of biomass carbon was obtained via the Calvin cycle during photoheterotrophic growth on acetate (101). Anaplerotic reactions during growth on other organic substrates could also contribute to the observed assimilation of DIC. Thus, while the high rate of DIC assimilation may be sufficient to support the growth of *R. globiformis* in its natural habitat, it is nevertheless unclear if and to what extent DIC assimilation is paired with light-driven assimilation of organic compounds. Regardless, the observed light-driven assimilation of DIC represents a significant amount of primary production by anoxygenic phototrophs in the Amphitheater spring, which are almost exclusively comprised of *R. globiformis*.

To examine the possibility of active photoheterotrophy, acetate was chosen as the organic substrate in light of its central role in carbon metabolism and the existence of measurable concentrations in many YNP hydrothermal fluids (74, 108). Rates of acetate assimilation in the light were over two orders of magnitude lower than rates of DIC assimilation in the light, and in contrast to DIC assimilation, rates of acetate assimilation were not different between microcosms incubated in the light and those excluded from light (Fig. 4), indicating that light-driven assimilation of acetate was not occurring in the spring when sampled. While it certainly remains possible that photoheterotrophy was occurring with other organic compounds, these results suggest that *R. globiformis* may execute photoautotrophy as its primary mode of carbon assimilation under typical conditions found at the Amphitheater spring. Nevertheless, it is plausible that rain events or other intermittent external processes may lead to spikes in DOC that would lead to an opportunistic temporary shift to photoheterotrophy, analogous to shifts between chemoautotrophy and chemoheterotrophy for which there is evidence in some hot springs in YNP (74, 109, 110). The DOC of acidic springs tends to be terrestrially derived (111, 112), consistent with intermittent surficial input of organic matter that could periodically drive photoheterotrophy. Both springs lie below marshy ground containing mosses and/or ferns and were also found to contain wood (Fig. 1), with the Amphitheater spring being bordered by a decaying log (presumably lodgepole pine, *Pinus contorta*). For example, this exogenous material could leach pentose sugars such as xylose into the spring waters via acid hydrolysis of pentosans such as xylan (8), consistent with *R. globiformis* possessing the genetic capacity for xylose metabolism (52).

A lack of light-driven assimilation of acetate may not be surprising given that growth of *R. globiformis* in culture was completely inhibited with acetate as the sole carbon source in early characterization studies by Pfennig (22). However, at the pH of the growth medium (5.6), a non-trivial portion (12.6%) of the acetate is speciated as acetic acid (Table S2). Growth medium was prepared by adding 1 g/L (12 mM) sodium acetate, resulting in a concentration of the neutral (protonated) acid of 1.5 mM at equilibrium. At such a concentration of the neutral species, the flux of acetic acid across the plasma membrane via passive diffusion may be detrimental to growth either by causing acidification of the cytoplasm upon deprotonation at the higher pH of the cytosol, forcing an unsustainable input of energy to maintain a suitable ΔpH across the plasma membrane (113), or due to toxicity of acetate itself (114, 115), thought to be caused by imbalances in essential metabolite concentrations created by efforts to counteract the increase in osmotic pressure (113). Regardless of mechanism, it may be that failure to observe growth on acetate was due to acetate toxicity rather than metabolic inability, the latter of which would be a notable shortcoming for a purple bacterium, as most purple bacteria can assimilate acetate (1, 116). All of the simple aliphatic carboxylates tested by Pfennig similarly inhibited growth, and only carboxylates where the calculated concentration of the neutral form was over an order of magnitude lower were found to support growth (Fig. S3). Thus, we hypothesized that in nature where total acetic acid + acetate concentrations are ~4 orders of magnitude less than those employed by Pfennig (0.83 µM in spring water; 1.4 µM in microcosms), despite being speciated more toward the protonated form (0.75 µM and 1.3 µM, respectively), *R. globiformis* may be capable of assimilating acetate into biomass by taking advantage of a manageable flux of acetic acid into the cytoplasm via passive diffusion. Indeed, there is evidence in the annotated genome (52) for the presence of acetate—CoA ligase, a key enzyme that would allow acetate to enter the tricarboxylic acid (TCA) cycle as acetyl-CoA, with carbon derived from acetate siphoned from the cycle as needed for anabolic processes. Replenishment of TCA cycle intermediates diverted to biosynthesis could be facilitated by the glyoxylate cycle (117), for which *R. globiformis* possesses the genetic capability (52). While it is possible that *R. globiformis* may lack one or more transport enzymes necessary for acetate metabolism, it is perhaps more plausible that for natural populations it is generally more efficient to take advantage of the significant DIC concentration of its environment (Table 1) and grow photoautotrophically, possibly via free membrane diffusion of aqueous carbon dioxide, the predominant form of DIC at low pH, rather than utilize much lower and perhaps dynamic concentrations of simple organic compounds for photoheterotrophic growth. It also remains possible *R. globiformis* may assimilate acetate independently of light, as chemotrophic growth in the dark at low O_2_ concentrations was reported with other organic substrates (22).

Acetate was dissimilated (oxidized to DIC) at a rate of ~15% of the rate of acetate assimilation, but only in microcosms excluded from light (Fig. S4), indicating that heterotrophs present in the Amphitheater mat only respired acetate in the dark. In comparison to rates of acetate metabolism at higher temperatures where phototrophs are excluded (74), the microbial community at the Amphitheater site exhibited a greater rate of assimilation relative to the rate of dissimilation than most hot springs examined, in particular when considering acidic springs (Fig. S5). On a dry mass basis, the acetate transformation rates are several orders of magnitude greater than those observed in high-temperature locations, which is undoubtedly due to the significantly larger amount of biomass per gram at the Amphitheater site than exists in the silica-rich sediments found in typical hot spring locations hosting chemosynthetic microbial communities.

### Geochemical habitat

Physicochemical data were obtained to characterize the habitat of *R. globiformis* and the biomass present in the mat, which are compiled in Table 1. The Amphitheater spring fluids are acidic with moderate concentrations of sulfate and little dissolved chloride. These observations indicate the spring waters are derived from meteoric water with input of volcanic gases containing hydrogen sulfide, like other acidic springs in YNP, with little to no input of deeply sourced hydrothermal waters (49). The stable isotope ratios of H_2_O from the spring lie close to the local meteoric water line, only slightly shifted due to modest amounts of evaporation (Fig. S6). The pH values reported here are consistent with the limited previous observations of other YNP springs where *R. globiformis* has been observed (27). The pH of the Hot Spring Basin spring measured in the field may be erroneous, as suggested by charge balance calculations, presumably due to the probe being inadequately submerged in the extremely shallow (~1 cm) water and/or contacting spring sediments. The pH calculated on the basis of achieving charge balance suggests that the lower pH limit for *R. globiformis* is well below 3. In spite of a reported pH range for growth of 4.2–6.5 when grown photoheterotrophically (22, 27), clearly, *R. globiformis* is capable of at least photoautotrophic growth under more acidic conditions. The temperatures observed at the springs were below 40°C, the reported upper temperature limit, but in excess of the optimum temperature range observed for photoheterotrophic growth in culture (22, 27). 16S rRNA gene sequences affiliated with *R. globiformis* were previously obtained from locations in the Sylvan Springs area with much higher temperatures at the time of sampling (47.2°C and 49.1°C [12]), indicating that either the thermotolerance of at least some strains of *R. globiformis* is much greater than previously observed, or that the sequences were derived from inactive and/or exogenous cells, perhaps originating from acidic sinter soils surrounding the thermal waters.

The total dissolved sulfide concentration at the Amphitheater site in 2017 was 210 µM, the highest observed in this study (Table 1). Initial growth studies indicated that sulfide completely inhibited growth at low concentrations, yet the lowest concentration tested was~0.4 mM (0.01%, assumed to refer to Na_2_S∙9H_2_O [22, 48]), roughly twice the highest concentration observed in the springs. While it is possible sulfide concentrations are maintained at lower concentrations in the mat than in the overlying spring waters due to biological sulfide oxidation, it appears that *R. globiformis* can tolerate sulfide at least in the ~0.1 mM range. Indeed, a strain of *Rhodopila* isolated from Lassen Volcanic National Park was grown in a liquid culture medium containing 0.14 mM sulfide (45). It remains an open question, however, whether *R. globiformis* is capable of utilizing sulfide either as an assimilatory sulfur source or as an electron donor for photosynthesis. In culture, thiosulfate has typically been employed as a sulfur source but was found to largely be oxidized to tetrathionate, indicating *R. globiformis* is capable of dissimilatory thiosulfate oxidation, though it was questioned whether this was sufficient to support photoautotrophy (118). Under acidic conditions (pH < 6), thiosulfate disproportionates to elemental sulfur and sulfite (46), which would be expected to maintain low thiosulfate concentrations in nature that may be insufficient to serve as an assimilatory source of sulfur or as an electron donor. Alternatively, sulfate can serve as a sulfur source at concentrations <1 mM (48), but the concentrations of sulfate observed in springs hosting *R. globiformis* in this study are equal to or exceed 1 mM (Table 1), suggesting sulfate may also prove inadequate as an assimilatory sulfur source in nature. Though its genome appears to lack a complete *sox* pathway for sulfide oxidation (52), a preponderance of the above nevertheless indicates that the metabolic role of sulfide in *R. globiformis* merits attention. Alternatively, hydrogen could potentially serve as a reducing agent for photosynthesis, with all genes encoding for a Ni-Fe hydrogenase being present in the genome of the type species (52). Though not measured in this study, a moderate concentration of hydrogen was measured in gases emanating from a frying pan at Amphitheater springs (0.37 mol% [85, 86]), thus it is plausible that a metabolically relevant aqueous concentration of hydrogen exists at the Amphitheater spring hosting *Rhodopila*.

As noted previously, the springs feature plentiful DIC that *R. globiformis* appears to exploit for photoautotrophy. The carbon isotopic ratio (δ^13^C) of the Amphitheater biomass was −26‰ in both years of sampling (Table 1). Relative to the isotopic composition of the DIC (−4‰), these ratios represent a fractionation (Δ^13^C) of −22‰, which is consistent with biomass synthesis via the reductive pentose phosphate (Calvin) cycle (119, 120), being very close to the fractionation during photoautotrophic growth via the Calvin cycle in the purple sulfur bacterium *Thermochromatium tepidum* (−20.5‰ [121]). As the DIC is speciated in essence completely as aqueous carbon dioxide due to the acidic pH (99.7% of the DIC), it is possible that *R. globiformis* takes advantage of passive membrane diffusion of aqueous carbon dioxide, which is the DIC uptake mechanism of the acidophilic alga *C. merolae* (122). Isotopic ratios of mat nitrogen were slightly more variable and are more difficult to interpret without isotopic data of potential nitrogen substrates, though they are consistent with those of other acidic hot springs in Yellowstone (112, 123, 124), where fluids are enriched in isotopically light ammonium putatively derived from input of vapor-phase gases from the hydrothermal system (123, 124). Both springs have ammonium concentrations that are unlikely to be limiting for ammonium assimilation by *R. globiformis* (Table 1), its preferred nitrogen source in culture (125). Though it has the genetic capacity for both ammonium assimilation and N_2_ fixation (52), diazotrophy is repressed in the presence of non-limiting ammonium concentrations (125).

**Fig 6:**
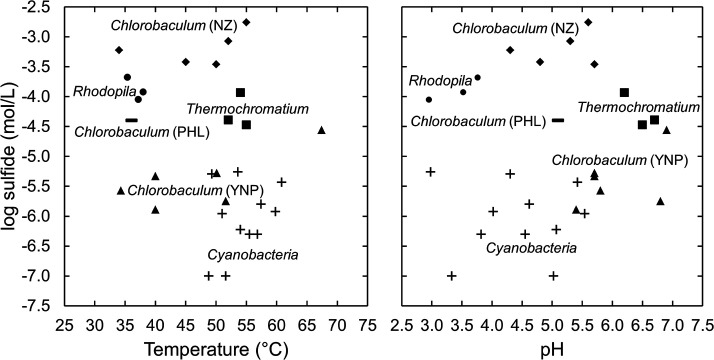
The logarithm of the total dissolved sulfide concentration versus temperature (left) and pH (right) of selected moderately acidic to circumneutral springs in Yellowstone where the predominant phototrophs are from the genus *Rhodopila* (●; this study), the genus *Thermochromatium* (■; 121, 126), the phylum *Cyanobacteria* (+; 7), or the genus *Chlorobaculum* (▲, 127). Locations with *Chlorobaculum* mats in New Zealand (♦, 128) and the Philippines (▬, 127) are also included. In cases where ranges are reported for sites with *Chlorobaculum*, the highest sulfide concentration, the highest temperature, and the lowest pH are plotted.

The springs in this study are geochemically similar to moderately acidic hot springs that host *Cyanobacteria* and algae (7), but are distinct in their lower temperatures and moderate concentrations of sulfide (Table 1; Fig. 6). In both cases, the moderately acidic pH values and moderate temperatures suggest a larger ratio of meteoric water to volcanic gas (vapor phase of the hydrothermal system) when compared to other acidic hot springs in YNP (7). Here, the persistence of sulfide in the spring water suggests that the injection of volcanic gases into oxygenated groundwater occurs very close to the surface, leading to the incomplete oxidation of sulfide. The modest temperatures of the springs additionally might indicate that the hydrothermal gases have cooled somewhat prior to mixing with groundwater. Both sites occur on the edges of thermal areas and beneath abundant terrestrial vegetation, indicative of cold nearby ground temperatures. The acidic spring waters do show evidence of some water-rock/soil reaction, given the major cation concentrations (Table 1). At the Amphitheater site, the major ion data indicate the spring water was more dilute in 2017 than in 2014, perhaps indicating a lower fluid flux or deeper input of gases in 2014 that would allow more time for sulfide oxidation and water-rock reactions, resulting in a lower sulfide concentration, lower pH, higher specific conductance, and slightly higher major ion concentrations.

The aforementioned purple sulfur bacterium *T. tepidum* (originally *Chromatium tepidum* [129]) has been studied in (and originally isolated from) springs in the Mammoth area in northern YNP with sulfide concentrations similar to those observed here (Fig. 6) (121, 126). Relative to *R. globiformis*, this organism is characterized by greater thermotolerance, being found in springs with temperatures of 44°C–55°C (121, 126, 130) and exhibiting a maximum temperature of 57°C in culture (130). These springs are also of higher pH, with values of 6.2–6.7 (121, 126), and the optimum pH of *T. tepidum* in culture is 7, though the pH range for growth has not been reported (130). The pH of spring waters at Mammoth is controlled by subsurface water-rock reactions with limestone, leading to fluids supersaturated with carbon dioxide that precipitate carbonates (travertine) upon degassing of carbon dioxide at the surface (131). Thus, these spring waters are buffered against significant decreases in pH resulting from the oxidation of sulfide, but depending on the extent of acid tolerance of *T. tepidum*, it is possible that springs with pH values <6 similar to those hosting *R. globiformis* but of somewhat higher temperature that could be habitable to *T. tepidum* may exist elsewhere in YNP. Thus, temperature and perhaps pH would dictate whether a moderately acidic spring with moderate levels of sulfide is more favorable to *R. globiformis* or *T. tepidum*. The green sulfur bacterium *Chlorobaculum tepidum* (previously *Chlorobium tepidum*; 132) inhabits waters with concentrations of dissolved sulfide of 0.3–1.8 mM in hot springs where it has been studied in New Zealand (Fig. 6) (128), though *Chlorobaculum* spp. in YNP were found in springs with low sulfide concentrations, similar to sulfide concentrations in springs hosting *Cyanobacteria* (Fig. 6) (127). However, YNP strains were successfully enriched in media containing 1 mM sulfide, indicating that if springs with high (~mM) concentrations of sulfide are identified in YNP, such conditions may exceed the tolerances of either the aforementioned purple bacteria (*R. globiformis* and *T. tepidum*) and could select for *Chlorobaculum*.

Though we report on the observation of springs featuring *Rhodopila* as the predominant anoxygenic phototroph in two additional thermal areas in YNP, springs with physicochemical conditions like those characterized here sustaining populations of *R. globiformis* nevertheless still appear to be relatively rare, such that it is essentially an endangered species (27). Important physicochemical components of the niche space for *R. globiformis* seem to include modest temperatures (30°C–40°C), acidic pH (<4), and moderate dissolved sulfide (~0.1 mM). Acidic springs may be found in YNP from ambient temperatures up to boiling (133), but the hydrological conditions allowing for a relatively small flux of hydrothermal gases into groundwater very close to the surface, resulting in moderate sulfide concentrations in springs only slightly elevated above ambient temperature, are apparently comparatively uncommon. Claims of low concentrations of sulfide being inhibitory for the growth of *R. globiformis* notwithstanding, moderate sulfide concentrations instead appear to be essential for the establishment of abundant natural populations. A continuous flux of hydrogen sulfide consumes dissolved oxygen in oxidation reactions and thereby maintains the low oxygen concentrations necessary for anaerobic or microaerobic phototrophic growth that otherwise may be exceeded due to the influx of atmospheric oxygen. Similarly, sulfide oxidation may quench oxygen produced in the spring by algae, the presence of which is indicated by 18S rRNA gene sequences (Fig. 2) and detection of chlorophyll *a* and the carotenoids diatoxanthin and fucoxanthin (Fig. 3), though it is unclear if and to what extent these algae are active in the spring and whether they are autochthonous. Additionally, the observed sulfide concentrations likely exceed the tolerances of at least some acidophilic algae, such as *C. merolae*, due to sulfide inhibition of electron transfer associated with photosystem II (10, 134), thereby freeing *R. globiformis* of some degree of interspecific competition for photo(auto)trophic niche space.

### Conclusions

Since its isolation roughly 50 years ago, *R. globiformis* has undergone numerous culture-dependent investigations revealing interesting features of its biochemistry, but knowledge of its ecology has not similarly expanded over that time, previously limited to brief reports of the isolation of similar organisms from new locations. In this work, we more thoroughly characterized the physicochemical conditions of its natural habitat and conducted carbon uptake experiments in the field to observe aspects of its physiology *in situ*. As purple nonsulfur bacteria are thought to favor and excel at photoheterotrophic growth, they were originally isolated and have been exclusively grown in the lab with organic substrates. The rate measurements here demonstrate that in its natural environment, it is responsible for a significant amount of primary production, utilizing the abundant supply of aqueous carbon dioxide offered by the flux of volcanic gases (i.e., magmatic CO_2_) into the spring waters, though to what extent this is combined with assimilation of organic compounds remains unclear. To our knowledge, the pH of the Amphitheater spring is the lowest at which light-driven DIC assimilation has been observed in a member of the domain Bacteria.

## Data Availability

Raw sequences were submitted to the NCBI Sequence Read Archive under BioProject ID PRJNA1174223.

## SUPPLEMENTAL MATERIAL

Supplemental material aem.01217-25-s0001.pdf Figures S1 to S6, Tables S1 to S5, and photographs of the Amphitheater spring.

ASM does not own the copyrights to Supplemental Material that may be linked to, or accessed through, an article. The authors have granted ASM a non-exclusive, world-wide license to publish the Supplemental Material files. Please contact the corresponding author directly for reuse.

## References

[1] Madigan MT, Jung DO. 2009. An overview of purple bacteria: systematics, physiology, and habitats. Adv Photosynth Respir 28:1–15.

[2] Madigan MT. 2003. Anoxygenic phototrophic bacteria from extreme environments. Photosynth Res 76:157–171. doi:10.1023/A:1024998212684 16228575

[3] Imhoff JF. 2017. Anoxygenic phototrophic bacteria from extreme environments, p 427–480. In Hallenbeck PC (ed), Modern topics in the phototrophic prokaryotes: environmental and applied aspects. Springer International Publishing.

[4] Colman DR, Poudel S, Hamilton TL, Havig JR, Selensky MJ, Shock EL, Boyd ES. 2018. Geobiological feedbacks and the evolution of thermoacidophiles. ISME J 12:225–236. doi:10.1038/ismej.2017.162 29028004 PMC5739016

[5] Colman DR, Keller LM, Arteaga-Pozo E, Andrade-Barahona E, St Clair B, Shoemaker A, Cox A, Boyd ES. 2024. Covariation of hot spring geochemistry with microbial genomic diversity, function, and evolution. Nat Commun 15:7506. doi:10.1038/s41467-024-51841-5 39209850 PMC11362583

[6] Brock TD. 1973. Lower pH limit for the existence of blue-green algae: evolutionary and ecological implications. Science 179:480–483. doi:10.1126/science.179.4072.480 4196167

[7] Fecteau KM, Boyd ES, Lindsay MR, Amenabar MJ, Robinson KJ, Debes RV II, Shock EL. 2022. Cyanobacteria and algae meet at the limits of their habitat ranges in moderately acidic hot springs. JGR Biogeosciences 127:e2021JG006446. doi:10.1029/2021JG006446

[8] Brock TD. 1978. Thermophilic microorganisms and life at high temperatures. Springer-Verlag, New York.

[9] Weeks K, Trembath-Reichert E, Boyer G, Fecteau K, Howells A, De Martini F, Gile GH, Shock EL. 2023. Characterization of microbiomic and geochemical compositions across the photosynthetic fringe. Front Microbiol 14:1176606. doi:10.3389/fmicb.2023.1176606 37187542 PMC10178925

[10] Boyd ES, Fecteau KM, Havig JR, Shock EL, Peters JW. 2012. Modeling the habitat range of phototrophs in Yellowstone National Park: toward the development of a comprehensive fitness landscape. Front Microbio 3:221. doi:10.3389/fmicb.2012.00221 PMC3376417 22719737

[11] Lynn R, Brock TD. 1969. Ecology of a species of Zygogonium in Yellowstone National Park. J Phycol 5:181–185. doi:10.1111/j.1529-8817.1969.tb02600.x 27096335

[12] Hamilton TL, Bennett AC, Murugapiran SK, Havig JR. 2019. Anoxygenic phototrophs span geochemical gradients and diverse morphologies in terrestrial geothermal springs. mSystems 4:e00498-19. doi:10.1128/mSystems.00498-19 31690593 PMC6832021

[13] Yurkov VV, Beatty JT. 1998. Aerobic anoxygenic phototrophic bacteria. Microbiol Mol Biol Rev 62:695–724. doi:10.1128/MMBR.62.3.695-724.1998 9729607 PMC98932

[14] Rathgeber C, Beatty JT, Yurkov V. 2004. Aerobic phototrophic bacteria: new evidence for the diversity, ecological importance and applied potential of this previously overlooked group. Photosyn Res 81:113–128. doi:10.1023/B:PRES.0000035036.49977.bc

[15] Imhoff JF. 2001. Transfer of Rhodopseudomonas acidophila to the new genus Rhodoblastus as Rhodoblastus acidophilus gen. nov., comb. nov. Int J Syst Evol Microbiol 51:1863–1866. doi:10.1099/00207713-51-5-1863 11594619

[16] Pfennig N. 1969. Rhodopseudomonas acidophila, sp. n., a new species of the budding purple nonsulfur bacteria. J Bacteriol 99:597–602. doi:10.1128/jb.99.2.597-602.1969 5821103 PMC250060

[17] Kempher ML, Madigan MT. 2012. Phylogeny and photoheterotrophy in the acidophilic phototrophic purple bacterium Rhodoblastus acidophilus*.* Arch Microbiol 194:567–574. doi:10.1007/s00203-012-0790-5 22286926

[18] Kulichevskaya IS, Guzev VS, Gorlenko VM, Liesack W, Dedysh SN. 2006. Rhodoblastus sphagnicola sp. nov., a novel acidophilic purple non-sulfur bacterium from Sphagnum peat bog. Int J Syst Evol Microbiol 56:1397–1402. doi:10.1099/ijs.0.63962-0 16738120

[19] Okamura K, Hisada T, Kanbe T, Hiraishi A. 2009. Rhodovastum atsumiense gen. nov., sp. nov., a phototrophic alphaproteobacterium isolated from paddy soil. J Gen Appl Microbiol 55:43–50. doi:10.2323/jgam.55.43 19282632

[20] Salama DM, Meyer TE, Kyndt JA. 2020. Genome sequence of the acidophilic nonsulfur purple photosynthetic alphaproteobacterium Rhodovastum atsumiense, a divergent member of the Acetobacteraceae family. Microbiology Resour Announc 9:e01541–19. 10.1128/MRA.01541-19 PMC7005127 32029562

[21] Imhoff JF, Trüper HG, Pfennig N. 1984. Rearrangement of the species and genera of the phototrophic “purple nonsulfur bacteria”. Int J Syst Bacteriol 34:340–343. doi:10.1099/00207713-34-3-340

[22] Pfennig N. 1974. Rhodopseudomonas globiformis, sp. n., a new species of the Rhodospirillaceae. Arch Microbiol 100:197–206. doi:10.1007/BF00446317

[23] Wichlacz PL, Unz RF, Langworthy TA. 1986. Acidiphilium angustum sp. nov., Acidiphilium facilis sp. nov., and Acidiphilium rubrum sp. nov.: acidophilic heterotrophic bacteria isolated from acidic coal mine drainage. Int J Syst Bacteriol 36:197–201. doi:10.1099/00207713-36-2-197

[24] Wakao N, Nagasawa N, Matsuura T, Matsukura H, Matsumoto T, Hiraishi A, Sakurai Y, Shiota H. 1994. Acidiphilium multivorum sp. nov., an acidophilic chemoorganotrophic bacterium from pyritic acid mine drainage. J Gen Appl Microbiol 40:143–159. doi:10.2323/jgam.40.143

[25] Hamamura N, Olson SH, Ward DM, Inskeep WP. 2005. Diversity and functional analysis of bacterial communities associated with natural hydrocarbon seeps in acidic soils at rainbow springs, Yellowstone National Park. Appl Environ Microbiol 71:5943–5950. doi:10.1128/AEM.71.10.5943-5950.2005 16204508 PMC1265959

[26] Sievers M, Ludwig W, Teuber M. 1994. Phylogenetic positioning of Acetobacter, Gluconobacter, Rhodopila and Acidiphilium species as a branch of acidophilic bacteria in the α-subclass of proteobacteria based on 16S ribosomal DNA sequences. Syst Appl Microbiol 17:189–196. doi:10.1016/S0723-2020(11)80006-8

[27] Imhoff JF, Madigan MT. 2021. Rhodopila. Bergey’s manual of systematics of bacteria and archaea. Wiley.

[28] Ambler RP, Meyer TE, Kamen MD. 1993. Amino acid sequence of a high redox potential ferredoxin (HiPIP) from the purple phototrophic bacterium Rhodopila globiformis, which has the highest known redox potential of its class. Arch Biochem Biophys 306:215–222. doi:10.1006/abbi.1993.1503 8215406

[29] Meyer TE, Przysiecki CT, Watkins JA, Bhattacharyya A, Simondsen RP, Cusanovich MA, Tollin G. 1983. Correlation between rate constant for reduction and redox potential as a basis for systematic investigation of reaction mechanisms of electron transfer proteins. Proc Natl Acad Sci USA 80:6740–6744. doi:10.1073/pnas.80.22.6740 6580615 PMC390061

[30] Ambler RP, Meyer TE, Cusanovich MA, Kamen MD. 1987. The amino acid sequence of the cytochrome c2 from the phototrophic bacterium Rhodopseudomonas globiformis. Biochem J 246:115–120. doi:10.1042/bj2460115 2823792 PMC1148247

[31] Benning MM, Meyer TE, Holden HM. 1996. Molecular structure of a high potential cytochrome c2 isolated from Rhodopila globiformis. Arch Biochem Biophys 333:338–348. doi:10.1006/abbi.1996.0400 8809072

[32] Bertini I, Capozzi F, Luchinat C, Piccioli M. 1993. 1H-NMR investigation of oxidized and reduced high-potential iron-sulfur protein from Rhodopseudomonas globiformis. Eur J Biochem 212:69–78. doi:10.1111/j.1432-1033.1993.tb17634.x 8444166

[33] Heering HA, Bulsink YBM, Hagen WR, Meyer TE. 1995. Reversible super-reduction of the cubane [4Fe-4S](3+;2+;1+) in the high-potential iron-sulfur protein under non-denaturing conditions. EPR spectroscopic and electrochemical studies. Eur J Biochem 232:811–817. doi:10.1111/j.1432-1033.1995.tb20877.x 7588720

[34] Heering HA, Bulsink BM, Hagen WR, Meyer TE. 1995. Influence of charge and polarity on the redox potentials of high-potential iron-sulfur proteins: evidence for the existence of two groups. Biochemistry 34:14675–14686. doi:10.1021/bi00045a008 7578075

[35] Menin L, Gaillard J, Parot P, Schoepp B, Nitschke W, Verméglio A. 1998. Role of HiPIP as electron donor to the RC-bound cytochrome in photosynthetic purple bacteria. Photosyn Res 55:343–348. doi:10.1023/A:1005989900756

[36] Przysiecki CT, Meyer TE, Cusanovich MA. 1985. Circular dichroism and redox properties of high redox potential ferredoxins. Biochemistry 24:2542–2549. doi:10.1021/bi00331a022 3925987

[37] Künzler A, Pfennig N. 1973. Das vorkommen von bacteriochlorophyll aP und aGg in stämmen aller arten der Rhodospirillaceae. Archiv Mikrobiol 91:83–86. doi:10.1007/BF00409541 4197122

[38] Schmidt K, Liaaen-Jensen S. 1973. Bacterial carotenoids. XLII. New keto-carotenoids from Rhodopseudomonas globiformis (Rhodospirillaceae). Acta Chem Scand 27:3040–3052. doi:10.3891/acta.chem.scand.27-3040 4778251

[39] Britton G, Liaaen-Jensen S, Pfander H, eds. 2004. Carotenoids handbook. Springer Basel AG.

[40] Tani K, Kanno R, Kurosawa K, Takaichi S, Nagashima KVP, Hall M, Yu LJ, Kimura Y, Madigan MT, Mizoguchi A, Humbel BM, Wang-Otomo ZY. 2022. An LH1-RC photocomplex from an extremophilic phototroph provides insight into origins of two photosynthesis proteins. Commun Biol 5:1197. doi:10.1038/s42003-022-04174-2 36344631 PMC9640540

[41] Kato S-I, Urakami T, Komagata K. 1985. Quinone systems and cellular fatty acid composition in species of Rhodospirillaceae genera. J Gen Appl Microbiol 31:381–398. doi:10.2323/jgam.31.381

[42] Hiraishi A, Hoshino Y, Kitamura H. 1984. Isoprenoid quinone composition in the classification of Rhodospirillaceae. J Gen Appl Microbiol 30:197–210. doi:10.2323/jgam.30.197

[43] Hiraishi A, Hoshino Y. 1984. Distribution of rhodoquinone in Rhodospirillaceae and its taxonomic implications. J Gen Appl Microbiol 30:435–448. doi:10.2323/jgam.30.435

[44] Pietsch K, Weckesser J, Fischer U, Mayer H. 1990. The lipopolysaccharides of Rhodospirillum rubrum, Rhodospirillum molischianum, and Rhodopila globiformis. Arch Microbiol 154:433–437. doi:10.1007/BF00245223

[45] Mayer MH, Parenteau MN, Kempher ML, Madigan MT, Jahnke LL, Welander PV. 2021. Anaerobic 3-methylhopanoid production by an acidophilic photosynthetic purple bacterium. Arch Microbiol 203:6041–6052. doi:10.1007/s00203-021-02561-7 34528111 PMC8590665

[46] Xu Y, Schoonen MAA, Nordstrom DK, Cunningham KM, Ball JW. 1998. Sulfur redox chemistry and the origin of thiosulfate in hydrothermal waters of Yellowstone National Park. Geochim Cosmochim Acta 62:3729–3743. doi:10.1016/S0016-7037(98)00269-5

[47] Cox JS, Holland ME, Shock EL. 2004. Dissolved free amino acids in hydrothermal springs at Yellowstone National Park. AGU Fall Meeting Abstracts. p B21B–0888, U.S.A

[48] Imhoff JF, Then J, Hashwa F, Trüper HG. 1981. Sulfate assimilation in Rhodopseudomonas globiformis. Arch Microbiol 130:234–237. doi:10.1007/BF00459525

[49] Nordstrom DK, McCleskey RB, Ball JW. 2009. Sulfur geochemistry of hydrothermal waters in Yellowstone National Park: IV Acid–sulfate waters. Appl Geochem 24:191–207. doi:10.1016/j.apgeochem.2008.11.019

[50] Sims KWW, Messa CM, Scott SR, Parsekian AD, Miller A, Role AL, Moloney TP, Shock EL, Lowenstern JB, McCleskey RB, Charette MA, Carr BJ, Pasquet S, Heasler H, Jaworowoski C, Holbrook WS, Lindsay MR, Colman DR, Boyd ES. 2023. The dynamic influence of subsurface geological processes on the assembly and diversification of thermophilic microbial communities in continental hydrothermal systems. Geochim Cosmochim Acta 362:77–103. doi:10.1016/j.gca.2023.10.021

[51] Fernandes-Martins MC, Colman DR, Boyd ES. 2024. Sulfide oxidation by members of the Sulfolobales. PNAS Nexus 3:pgae201. doi:10.1093/pnasnexus/pgae201 PMC11143483 38827816

[52] Imhoff JF, Rahn T, Künzel S, Neulinger SC. 2018. New insights into the metabolic potential of the phototrophic purple bacterium Rhodopila globiformis DSM 161^T^ from its draft genome sequence and evidence for a vanadium-dependent nitrogenase. Arch Microbiol 200:847–857. doi:10.1007/s00203-018-1489-z 29423563

[53] Boyer GM, Schubotz F, Summons RE, Woods J, Shock EL. 2020. Carbon oxidation state in microbial polar lipids suggests adaptation to hot spring temperature and redox gradients. Front Microbiol 11:229. doi:10.3389/fmicb.2020.00229 32153529 PMC7044123

[54] St Clair B, Pottenger J, Debes R, Hanselmann K, Shock E. 2019. Distinguishing biotic and abiotic iron oxidation at low temperatures. ACS Earth Space Chem 3:905–921. doi:10.1021/acsearthspacechem.9b00016

[55] Robinson KJ. 2017. Modeling aqueous organic chemistry in experimental and natural systems. Ph.D. dissertation, Arizona State University

[56] Lindsay MR, Amenabar MJ, Fecteau KM, Debes RV, Fernandes Martins MC, Fristad KE, Xu H, Hoehler TM, Shock EL, Boyd ES. 2018. Subsurface processes influence oxidant availability and chemoautotrophic hydrogen metabolism in Yellowstone hot springs. Geobiology 16:674–692. doi:10.1111/gbi.12308 30035368

[57] Wolery TW, Jarek RL. 2003. Software user’s manual EQ3/6, Version 8.0. U.S. Department of Energy, Office of Civilian Radioactive Waste Management, Office of Repository Development.

[58] Shock EL, Sassani DC, Willis M, Sverjensky DA. 1997. Inorganic species in geologic fluids: correlations among standard molal thermodynamic properties of aqueous ions and hydroxide complexes. Geochim Cosmochim Acta 61:907–950. doi:10.1016/s0016-7037(96)00339-0 11541225

[59] Sverjensky DA, Shock EL, Helgeson HC. 1997. Prediction of the thermodynamic properties of aqueous metal complexes to 1000 degrees C and 5 kb. Geochim Cosmochim Acta 61:1359–1412. doi:10.1016/s0016-7037(97)00009-4 11541435

[60] Boyer G, Robare J, Ely T, Shock E. 2022. AqEquil: Python package for aqueous geochemical speciation (0.15.3). Zenodo. doi:10.5281/zenodo.6382932

[61] Hamilton TL, Peters JW, Skidmore ML, Boyd ES. 2013. Molecular evidence for an active endogenous microbiome beneath glacial ice. ISME J 7:1402–1412. doi:10.1038/ismej.2013.31 23486249 PMC3695297

[62] Yutin N, Suzuki MT, Rosenberg M, Rotem D, Madigan MT, Süling J, Imhoff JF, Béjà O. 2009. BchY-based degenerate primers target all types of anoxygenic photosynthetic bacteria in a single PCR. Appl Environ Microbiol 75:7556–7559. doi:10.1128/AEM.01014-09 19801482 PMC2786397

[63] Amaral-Zettler LA, McCliment EA, Ducklow HW, Huse SM. 2009. A method for studying protistan diversity using massively parallel sequencing of V9 hypervariable regions of small-subunit ribosomal RNA genes. PLoS One 4:e6372. doi:10.1371/journal.pone.0006372 19633714 PMC2711349

[64] Stoeck T, Bass D, Nebel M, Christen R, Jones MDM, Breiner HW, Richards TA. 2010. Multiple marker parallel tag environmental DNA sequencing reveals a highly complex eukaryotic community in marine anoxic water. Mol Ecol 19 Suppl 1:21–31. doi:10.1111/j.1365-294X.2009.04480.x 20331767

[65] Parada AE, Needham DM, Fuhrman JA. 2016. Every base matters: assessing small subunit rRNA primers for marine microbiomes with mock communities, time series and global field samples. Environ Microbiol 18:1403–1414. doi:10.1111/1462-2920.13023 26271760

[66] Andrews S. 2010. FastOC: a quality control tool for high throughput sequence data

[67] Callahan BJ, McMurdie PJ, Rosen MJ, Han AW, Johnson AJA, Holmes SP. 2016. DADA2: high-resolution sample inference from Illumina amplicon data. Nat Methods 13:581–583. doi:10.1038/nmeth.3869 27214047 PMC4927377

[68] Bolyen E, Rideout JR, Dillon MR, Bokulich NA, Abnet CC, Al-Ghalith GA, Alexander H, Alm EJ, Arumugam M, Asnicar F, et al.. 2019. Reproducible, interactive, scalable and extensible microbiome data science using QIIME 2. Nat Biotechnol 37:852–857. doi:10.1038/s41587-019-0209-9 31341288 PMC7015180

[69] Quast C, Pruesse E, Yilmaz P, Gerken J, Schweer T, Yarza P, Peplies J, Glöckner FO. 2013. The SILVA ribosomal RNA gene database project: improved data processing and web-based tools. Nucleic Acids Res 41:D590–6. doi:10.1093/nar/gks1219 23193283 PMC3531112

[70] R Core Team. 2023. R: a language and environment for statistical computing. Vienna, Austria

[71] Oksanen J, Simpson GL, Blanchet FG, Kindt R, Legendre P, Minchin PR, O’Hara RB, Solymos P, Stevens MHH, Szoecs E, et al.. 2022. vegan: community ecology package

[72] Sander LC, Sharpless KE, Craft NE, Wise SA. 1994. Development of engineered stationary phases for the separation of carotenoid isomers. Anal Chem 66:1667–1674. doi:10.1021/ac00082a012 15943024

[73] van Breemen RB, Dong L, Pajkovic ND. 2012. Atmospheric pressure chemical ionization tandem mass spectrometry of carotenoids. Int J Mass Spectrom 312:163–172. doi:10.1016/j.ijms.2011.07.030 22408388 PMC3293484

[74] Urschel MR, Kubo MD, Hoehler TM, Peters JW, Boyd ES. 2015. Carbon source preference in chemosynthetic hot spring communities. Appl Environ Microbiol 81:3834–3847. doi:10.1128/AEM.00511-15 25819970 PMC4421056

[75] Peterson BJ. 1980. Aquatic primary productivity and the 14C-CO2 method: a history of the productivity problem. Annu Rev Ecol Syst 11:359–385. doi:10.1146/annurev.es.11.110180.002043

[76] Mathur J, Bizzoco RW, Ellis DG, Lipson DA, Poole AW, Levine R, Kelley ST. 2007. Effects of abiotic factors on the phylogenetic diversity of bacterial communities in acidic thermal springs. Appl Environ Microbiol 73:2612–2623. doi:10.1128/AEM.02567-06 17220248 PMC1855587

[77] Doemel WN, Brock TD. 1970. The upper temperature limit of Cyanidium caldarium. Arch Mikrobiol 72:326–332. doi:10.1007/BF00409031 5474131

[78] Doemel WN, Brock TD. 1971. The physiological ecology of Cyanidium caldarium. J Gen Microbiol 67:17–32. doi:10.1099/00221287-67-1-17

[79] Skorupa DJ, Reeb V, Castenholz RW, Bhattacharya D, McDermott TR. 2013. Cyanidiales diversity in Yellowstone National Park. Lett Appl Microbiol 57:459–466. doi:10.1111/lam.12135 23865641

[80] Skorupa DJ, Castenholz RW, Mazurie A, Carey C, Rosenzweig F, McDermott TR. 2014. In situ gene expression profiling of the thermoacidophilic alga Cyanidioschyzon in relation to visible and ultraviolet irradiance. Environ Microbiol 16:1627–1641. doi:10.1111/1462-2920.12317 24274381

[81] Toplin JA, Norris TB, Lehr CR, McDermott TR, Castenholz RW. 2008. Biogeographic and phylogenetic diversity of thermoacidophilic cyanidiales in Yellowstone National Park, Japan, and New Zealand. Appl Environ Microbiol 74:2822–2833. doi:10.1128/AEM.02741-07 18344337 PMC2394875

[82] Stephens TG, Van Etten J, McDermott T, Christian W, Chaverra M, Gurney J, Lee Y, Kim H, Cho CH, Chovancek E, Westhoff P, Otte A, Northen TR, Bowen BP, Louie KB, Barry K, Grigoriev IV, Mock T, Liu S-L, Miyagishima S-Y, Yoshinaga M, Weber APM, Yoon HS, Bhattacharya D. 2024. Temporal dynamics in a red alga dominated geothermal feature in Yellowstone National Park. ISME Commun 4:ycae151. doi:10.1093/ismeco/ycae151 39711979 PMC11662350

[83] Hurwitz S, Evans WC, Lowenstern JB, Bergfeld D, Werner C, Heasler H, Jaworowski C. 2007. Extensive hydrothermal rock alteration in a low pH, steam-heated environment: Hot Springs Basin, Yellowstone National Park. Water-Rock Interact - Proc 12th Int Symp Water-Rock Interact WRI-12

[84] Werner C, Hurwitz S, Evans WC, Lowenstern JB, Bergfeld D, Heasler H, Jaworowski C, Hunt A. 2008. Volatile emissions and gas geochemistry of Hot Spring Basin, Yellowstone National Park, USA. Journal of Volcanology and Geothermal Research 178:751–762. doi:10.1016/j.jvolgeores.2008.09.016

[85] Bergfeld D, Lowenstern JB, Hunt AG, Shanks W, Evans WC. 2014. Gas and isotope chemistry of thermal features in Yellowstone National Park, Wyoming (ver. 1.1). US Geol Surv Sci Investig Rep

[86] Bergfeld D, Lowenstern J, Hunt A, Hurwitz S, McCleskey B, Peek S. 2019. Chemical and isotopic data on gases and waters for thermal and non-thermal features across Yellowstone National Park (ver. 2.0, March 2019). US Geol Surv data release. doi:10.5066/F7H13105

[87] Moreira D, Amils R. 1997. Phylogeny of Thiobacillus cuprinus and other mixotrophic thiobacilli: proposal for Thiomonas gen. nov. Int J Syst Bacteriol 47:522–528. doi:10.1099/00207713-47-2-522 9103643

[88] Arsène-Ploetze F, Koechler S, Marchal M, Coppée J-Y, Chandler M, Bonnefoy V, Brochier-Armanet C, Barakat M, Barbe V, Battaglia-Brunet F, et al.. 2010. Structure, function, and evolution of the Thiomonas spp. genome. PLoS Genet 6:e1000859. doi:10.1371/journal.pgen.1000859 20195515 PMC2829063

[89] Inskeep WP, Macur RE, Harrison G, Bostick BC, Fendorf S. 2004. Biomineralization of As(V)-hydrous ferric oxyhydroxide in microbial mats of an acid-sulfate-chloride geothermal spring, Yellowstone National Park. Geochim Cosmochim Acta 68:3141–3155. doi:10.1016/j.gca.2003.09.020

[90] Macur RE, Langner HW, Kocar BD, Inskeep WP. 2004. Linking geochemical processes with microbial community analysis: successional dynamics in an arsenic-rich, acid-sulphatec-hloride geothermal spring. Geobiology 2:163–177. doi:10.1111/j.1472-4677.2004.00032.x

[91] Stohr R, Waberski A, Völker H, Tindall BJ, Thomm M. 2001. Hydrogenothermus marinus gen. nov., sp. nov., a novel thermophilic hydrogen-oxidizing bacterium, recognition of Calderobacterium hydrogenophilum as a member of the genus Hydrogenobacter and proposal of the reclassification of Hydrogenobacter acidophilus as Hydrogenobaculum acidophilum gen. nov., comb. nov., in the phylum “Hydrogenobacter/Aquifex”. Int J Syst Evol Microbiol 51:1853–1862. doi:10.1099/00207713-51-5-1853 11594618

[92] Stieglmeier M, Klingl A, Alves RJE, Rittmann SK-MR, Melcher M, Leisch N, Schleper C. 2014. Nitrososphaera viennensis gen. nov., sp. nov., an aerobic and mesophilic, ammonia-oxidizing archaeon from soil and a member of the archaeal phylum Thaumarchaeota. Int J Syst Evol Microbiol 64:2738–2752. doi:10.1099/ijs.0.063172-0 24907263 PMC4129164

[93] Pennington F, Guillard RRL, Liaaen-Jensen S. 1988. Carotenoid distribution patterns in Bacillariophyceae (Diatoms). Biochem Syst Ecol 16:589–592. doi:10.1016/0305-1978(88)90067-1

[94] Takaichi S. 2011. Carotenoids in algae: distributions, biosyntheses and functions. Mar Drugs 9:1101–1118. doi:10.3390/md9061101 21747749 PMC3131562

[95] Wakao N, Yokoi N, Isoyama N, Hiraishi A, Shimada K, Kobayashi M, Kise H, Iwaki M, Itoh S, Takaichi S, Sakurai Y. 1996. Discovery of natural photosynthesis using Zn-containing bacteriochlorophyll in an aerobic bacterium Acidiphilium rubrum. Plant and Cell Physiology 37:889–893. doi:10.1093/oxfordjournals.pcp.a029029

[96] Hiraishi A, Shimada K. 2001. Aerobic anoxygenic photosynthetic bacteria with zinc-bacteriochlorophyll. J Gen Appl Microbiol 47:161–180. doi:10.2323/jgam.47.161 12483616

[97] Kobayashi M, Yamamura M, Akiyama M, Kise H, Inoue K, Hara M, Wakao N, Yahara K, Watanabe T. 1998. Acid resistance of Zn-bacteriochlorophyll a from an acidophilic bacterium Acidiphilium rubrum. Anal Sci 14:1149–1152. doi:10.2116/analsci.14.1149

[98] Lehr CR, Frank SD, Norris TB, D’Imperio S, Kalinin AV, Toplin JA, Castenholz RW, McDermott TR. 2007. Cyanidia (cyanidiales) population diversity and dynamics in an acid-sulfate-chloride spring in yellowstone national park. J Phycol 43:3–14. doi:10.1111/j.1529-8817.2006.00293.x

[99] Erb TJ. 2011. Carboxylases in natural and synthetic microbial pathways. Appl Environ Microbiol 77:8466–8477. doi:10.1128/AEM.05702-11 22003013 PMC3233076

[100] Alloul A, Blansaer N, Cabecas Segura P, Wattiez R, Vlaeminck SE, Leroy B. 2023. Dehazing redox homeostasis to foster purple bacteria biotechnology. Trends Biotechnol 41:106–119. doi:10.1016/j.tibtech.2022.06.010 35843758

[101] McKinlay JB, Harwood CS. 2010. Carbon dioxide fixation as a central redox cofactor recycling mechanism in bacteria. Proc Natl Acad Sci U S A 107:11669–11675. doi:10.1073/pnas.1006175107 20558750 PMC2900684

[102] Gordon GC, McKinlay JB. 2014. Calvin cycle mutants of photoheterotrophic purple nonsulfur bacteria fail to grow due to an electron imbalance rather than toxic metabolite accumulation. J Bacteriol 196:1231–1237. doi:10.1128/JB.01299-13 24415727 PMC3957710

[103] Laguna R, Tabita FR, Alber BE. 2011. Acetate-dependent photoheterotrophic growth and the differential requirement for the Calvin-Benson-Bassham reductive pentose phosphate cycle in Rhodobacter sphaeroides and Rhodopseudomonas palustris. Arch Microbiol 193:151–154. doi:10.1007/s00203-010-0652-y 21104179

[104] Schuler CG, Havig JR, Hamilton TL. 2017. Hot spring microbial community composition, morphology, and carbon fixation: implications for interpreting the ancient rock record. Front Earth Sci 5:97. doi:10.3389/feart.2017.00097

[105] Havig JR, Hamilton TL. 2019. Productivity and community composition of low biomass/high silica precipitation hot springs: a possible window to Earth’s early biosphere? Life 9:64. doi:10.3390/life9030064 31362401 PMC6789502

[106] Bennett AC, Murugapiran SK, Hamilton TL. 2020. Temperature impacts community structure and function of phototrophic Chloroflexi and Cyanobacteria in two alkaline hot springs in Yellowstone National Park. Environ Microbiol Rep 12:503–513. doi:10.1111/1758-2229.12863 32613733 PMC7540483

[107] Zhang Y, Qi X, Wang S, Wu G, Briggs BR, Jiang H, Dong H, Hou W. 2020. Carbon fixation by photosynthetic mats along a temperature gradient in a Tengchong hot spring. JGR Biogeosciences 125:1–13. doi:10.1029/2020JG00571936158138

[108] Windman T. 2010. Organic Compounds in Hydrothermal Systems. Ph.D. dissertation, Arizona State University

[109] Schubotz F, Meyer-Dombard DR, Bradley AS, Fredricks HF, Hinrichs KU, Shock EL, Summons RE. 2013. Spatial and temporal variability of biomarkers and microbial diversity reveal metabolic and community flexibility in streamer biofilm communities in the Lower Geyser Basin, Yellowstone National Park. Geobiology 11:549–569. doi:10.1111/gbi.12051 23981055

[110] Schubotz F, Hays LE, Meyer-Dombard DR, Gillespie A, Shock EL, Summons RE. 2015. Stable isotope labeling confirms mixotrophic nature of streamer biofilm communities at alkaline hot springs. Front Microbiol 6:1–18. doi:10.3389/fmicb.2015.00042 25699032 PMC4318418

[111] Nye JJ, Shock EL, Hartnett HE. 2020. A novel PARAFAC model for continental hot springs reveals unique dissolved organic carbon compositions. Org Geochem 141:103964. doi:10.1016/j.orggeochem.2019.103964

[112] Sturrup C, Szynkiewicz A. 2025. Evaluating bulk carbon and nitrogen isotope compositions of acidic hot spring and mudpot sediments as biosignatures. Journal of Volcanology and Geothermal Research 466:108402. doi:10.1016/j.jvolgeores.2025.108402

[113] Pinhal S, Ropers D, Geiselmann J, de Jong H. 2019. Acetate metabolism and the inhibition of bacterial growth by acetate. J Bacteriol 201:e00147-19. doi:10.1128/JB.00147-19 30988035 PMC6560135

[114] Russell JB. 1992. Another explanation for the toxicity of fermentation acids at low pH: anion accumulation versus uncoupling. Journal of Applied Bacteriology 73:363–370. doi:10.1111/j.1365-2672.1992.tb04990.x

[115] Russell JB, Diez-Gonzalez F. 1998. The effects of fermentation acids on bacterial growth. Adv Microb Physiol 39:205–234. doi:10.1016/s0065-2911(08)60017-x 9328648

[116] Tabita RF. 1995. The biochemistry and metabolic regulation of carbon metabolism and CO_2_ fixation in purple bacteria, p 885–914. In Blankenship RE, Madigan MT, Bauer CE (ed), Anoxygenic photosynthetic bacteria. Kluwer Academic Publishers.

[117] Tang KH, Tang YJ, Blankenship RE. 2011. Carbon metabolic pathways in phototrophic bacteria and their broader evolutionary implications. Front Microbiol 2:165. doi:10.3389/fmicb.2011.00165 21866228 PMC3149686

[118] Then J, Trüper HG. 1981. The role of thiosulfate in sulfur metabolism of Rhodopseudomonas globiformis. Arch Microbiol 130:143–146. doi:10.1007/BF00411067

[119] Havig JR, Raymond J, Meyer-Dombard DR, Zolotova N, Shock EL. 2011. Merging isotopes and community genomics in a siliceous sinter-depositing hot spring. J Geophys Res 116:1–15. doi:10.1029/2010JG001415

[120] Smith DA, Steele A, Bowden R, Fogel ML. 2015. Ecologically and geologically relevant isotope signatures of C, N, and S: okenone producing purple sulfur bacteria part I. Geobiology 13:278–291. doi:10.1111/gbi.12136 25857753

[121] Madigan MT, Takigiku R, Lee RG, Gest H, Hayes JM. 1989. Carbon isotope fractionation by thermophilic phototrophic sulfur bacteria: evidence for autotrophic growth in natural populations. Appl Environ Microbiol 55:639–644. doi:10.1128/aem.55.3.639-644.1989 11536609 PMC184172

[122] Zenvirth D, Volokita M, Kaplan A. 1985. Photosynthesis and inorganic carbon accumulation in the acidophilic alga Cyanidioschyzon merolae*.* Plant Physiol 77:237–239. doi:10.1104/pp.77.1.237 16664017 PMC1064490

[123] Holloway JAM, Nordstrom DK, Böhlke JK, McCleskey RB, Ball JW. 2011. Ammonium in thermal waters of Yellowstone National Park: processes affecting speciation and isotope fractionation. Geochim Cosmochim Acta 75:4611–4636. doi:10.1016/j.gca.2011.05.036

[124] Havig JR, Hamilton TL. 2025. Between a rock and a soft place: biomass δ^15^N values of Yellowstone hot spring microbial communities and their potential for preservation in the rock record. JGR Biogeosciences 130. doi:10.1029/2024JG008136

[125] Madigan M, Cox SS. 1982. Nitrogen metabolism in Rhodopseudomonas globiformis. Arch Microbiol 133:6–10. doi:10.1007/BF00943761

[126] Klatt CG, Inskeep WP, Herrgard MJ, Jay ZJ, Rusch DB, Tringe SG, Niki Parenteau M, Ward DM, Boomer SM, Bryant DA, Miller SR. 2013. Community structure and function of high-temperature chlorophototrophic microbial mats inhabiting diverse geothermal environments. Front Microbiol 4:106. doi:10.3389/fmicb.2013.00106 23761787 PMC3669762

[127] Bedard DL, Van Slyke G, Nübel U, Bateson MM, Brumfield S, An YJ, Becraft ED, Wood JM, Thiel V, Ward DM. 2023. Geographic and ecological diversity of green sulfur bacteria in hot spring mat communities. Microorganisms 11:2921. doi:10.3390/microorganisms11122921 38138064 PMC10746008

[128] Castenholz RW, Bauld J, Jørgenson BB. 1990. Anoxygenic microbial mats of hot springs: thermophilic Chlorobium sp. FEMS Microbiol Lett 74:325–336. doi:10.1111/j.1574-6968.1990.tb04079.x

[129] Imhoff JF, Süling J, Petri R. 1998. Phylogenetic relationships among the Chromatiaceae, their taxonomic reclassification and description of the new genera Allochromatium, Halochromatium, Isochromatium, Marichromatiurn, Thiococcus, Thiohalocapsa and Thermochromatium. Int J Syst Evol Microbiol 48:1129–1143. 10.1099/00207713-48-4-1129 9828415

[130] Madigan MT. 1986. Chromatium tepidum sp. nov., a thermophilic photosynthetic bacterium of the family Chromatiaceae. Int J Syst Bacteriol 36:222–227. doi:10.1099/00207713-36-2-222

[131] Fouke BW. 2011. Hot‐spring systems geobiology: abiotic and biotic influences on travertine formation at Mammoth hot-spring, Yellowstone National Park, USA. Sedimentology 58:170–219. doi:10.1111/j.1365-3091.2010.01209.x

[132] Imhoff JF. 2003. Phylogenetic taxonomy of the family Chlorobiaceae on the basis of 16S rRNA and fmo (Fenna-Matthews-Olson protein) gene sequences. Int J Syst Evol Microbiol 53:941–951. doi:10.1099/ijs.0.02403-0 12892110

[133] Amenabar MJ, Urschel MR, Boyd ES. 2015. Metabolic and taxonomic diversification in continental magmatic hydrothermal systems, p 57–96. In Bakermans C (ed), Microbial evolution under extreme conditions. De Gruyter.

[134] Cox A, Shock EL, Havig JR. 2011. The transition to microbial photosynthesis in hot spring ecosystems. Chem Geol 280:344–351. doi:10.1016/j.chemgeo.2010.11.022

