# Erratum for Fecteau et al., “Primary production by the purple nonsulfur bacterium *Rhodopila globiformis* in an acidic, moderately sulfidic warm spring”

**DOI:** 10.1128/aem.02509-25

**Published:** 2026-01-13

**Authors:** Kristopher M. Fecteau, Katelyn M. Weeks, R. Vincent Debes, Tanner J. Barnes, Kirtland J. Robinson, Joshua J. Nye, Melody R. Lindsay, Eric S. Boyd, Everett L. Shock

## ERRATUM

Volume 91, no. 10, e01217-25, 2025, https://doi.org/10.1128/aem.01217-25.

Page 6, line 45: “vegan package (v2.6-4; 71)” should read “vegan package (v.2.6-4 [71]).”

Page 8, line 41: “isotopic discrimination factor (1.05; 75)” should read “isotopic discrimination factor (1.05 [75]).”

Page 10, line 8: “under acidic conditions (~56°C; 77)” should read “under acidic conditions (~56°C [77]).”

Page 18, line 37: “As purple nonsulfur bacteria are thought to favor and excel at photoheterotrophic growth, they were originally isolated and have been exclusively grown in the lab with organic substrates.” should read “As purple nonsulfur bacteria are thought to favor and excel at photoheterotrophic growth, *R. globiformis* was originally isolated and has been exclusively grown in the lab with organic substrates.”

