# Supplementary material for: Primary production by the purple nonsulfur bacterium Rhodopila globiformis in an acidic, moderately sulfidic warm spring
Source: Appl Environ Microbiol. 2025 Sep 10;91(10):e01217-25. doi: 10.1128/aem.01217-25 (PMC12542754; doi:10.1128/aem.01217-25)
Supplement: Supplemental material — Figures S1 to S6, Tables S1 to S5, and photographs of the Amphitheater spring. [file aem.01217-25-s0001.pdf]

Supplemental Material

to accompany

Primary production by the purple nonsulfur bacterium *Rhodopila globiformis* in an acidic, moderately sulfidic warm spring

Kristopher M. Fecteau,[1,2] Katelyn M. Weeks,[2] R. Vincent Debes II,[1] Tanner J. Barnes,[2] Kirtland J. Robinson,[1] Joshua J. Nye,[2] Melody R. Lindsay,[3] Eric S. Boyd,[3] and Everett L. Shock[1,2]

[1]School of Earth and Space Exploration, Arizona State University, Tempe, Arizona 85287
[2]School of Molecular Sciences, Arizona State University, Tempe, Arizona 85287
[3]Department of Microbiology and Cell Biology, Montana State University, Bozeman, Montana 59717

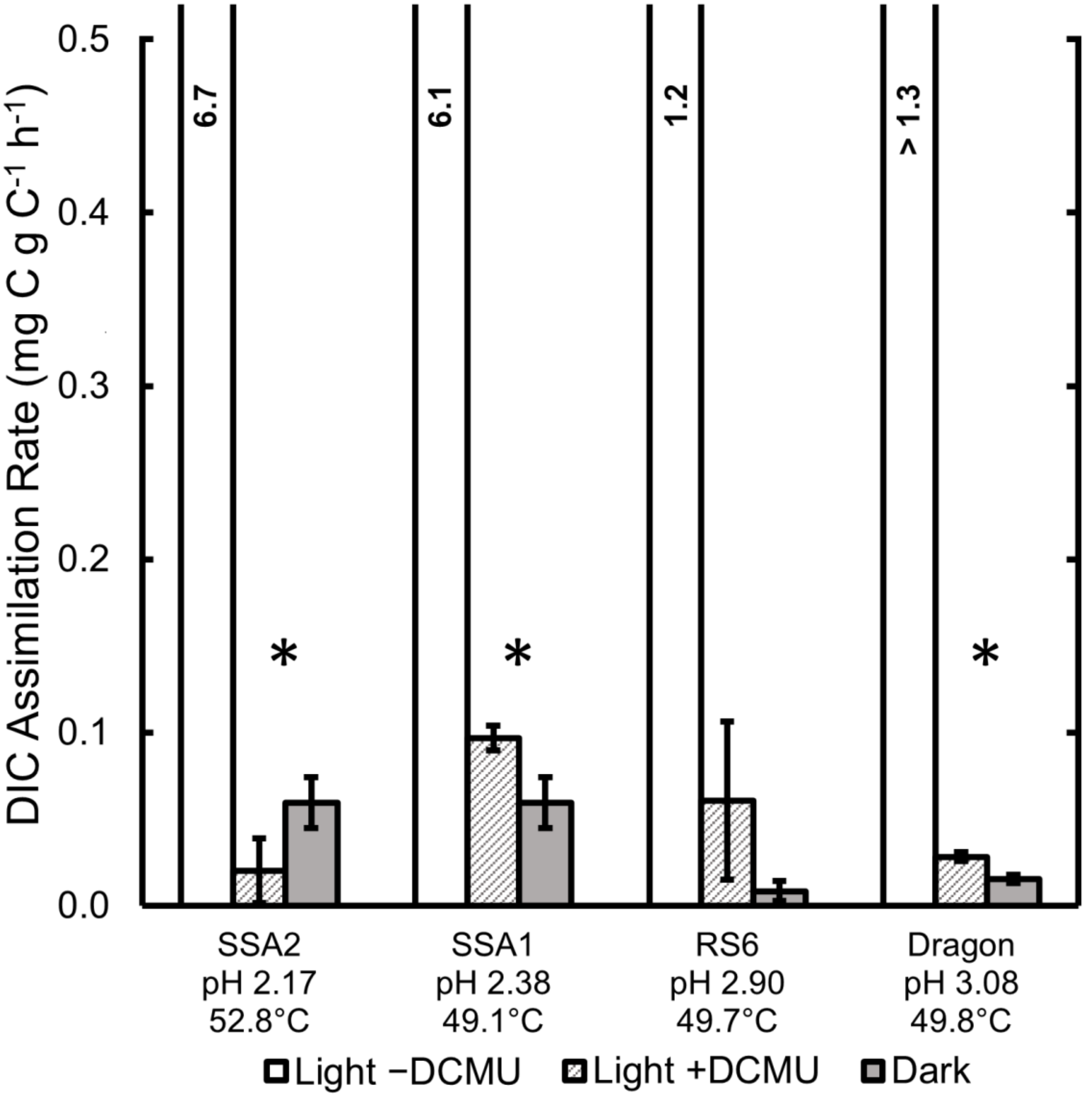

Figure S1. Rates of dissolved inorganic carbon (DIC) assimilation at additional acidic hot spring locations in Yellowstone National Park. Rates determined in microcosms exposed to light in the absence of 3-(3,4-dichlorophenyl)-1,1-dimethylurea (Light −DCMU; open bars) all exceeded 0.5 mg C g C^{-1} h^{-1} and are off-scale; rates are indicated numerically inside each bar. The rate for the outflow of Dragon Spring is a minimum value due to the activities in each microcosm exceeding the range of the scintillation counter. Rates determined in microcosms exposed to light and amended with DCMU (Light +DCMU; 10 μM final concentration; grey stripes) and rates determined in microcosms excluded from light (Dark; wrapped in aluminum foil; solid grey bars) are also shown; cases where the difference in rate between the Light +DCMU and Dark treatments is statistically significant (t-test, $p < 0.05$) are indicated with an asterisk. Data for SSA1 and SSA2 (Sylvan Springs Area, Gibbon Geyser Basin) were previously reported by Hamilton *et al.* (2019); rates for RS6 (Rabbit Creek thermal area) and for the outflow of Dragon Spring (Norris Geyser Basin) were determined as described in the Methods and have not been previously reported. All data are compiled in Table S4.

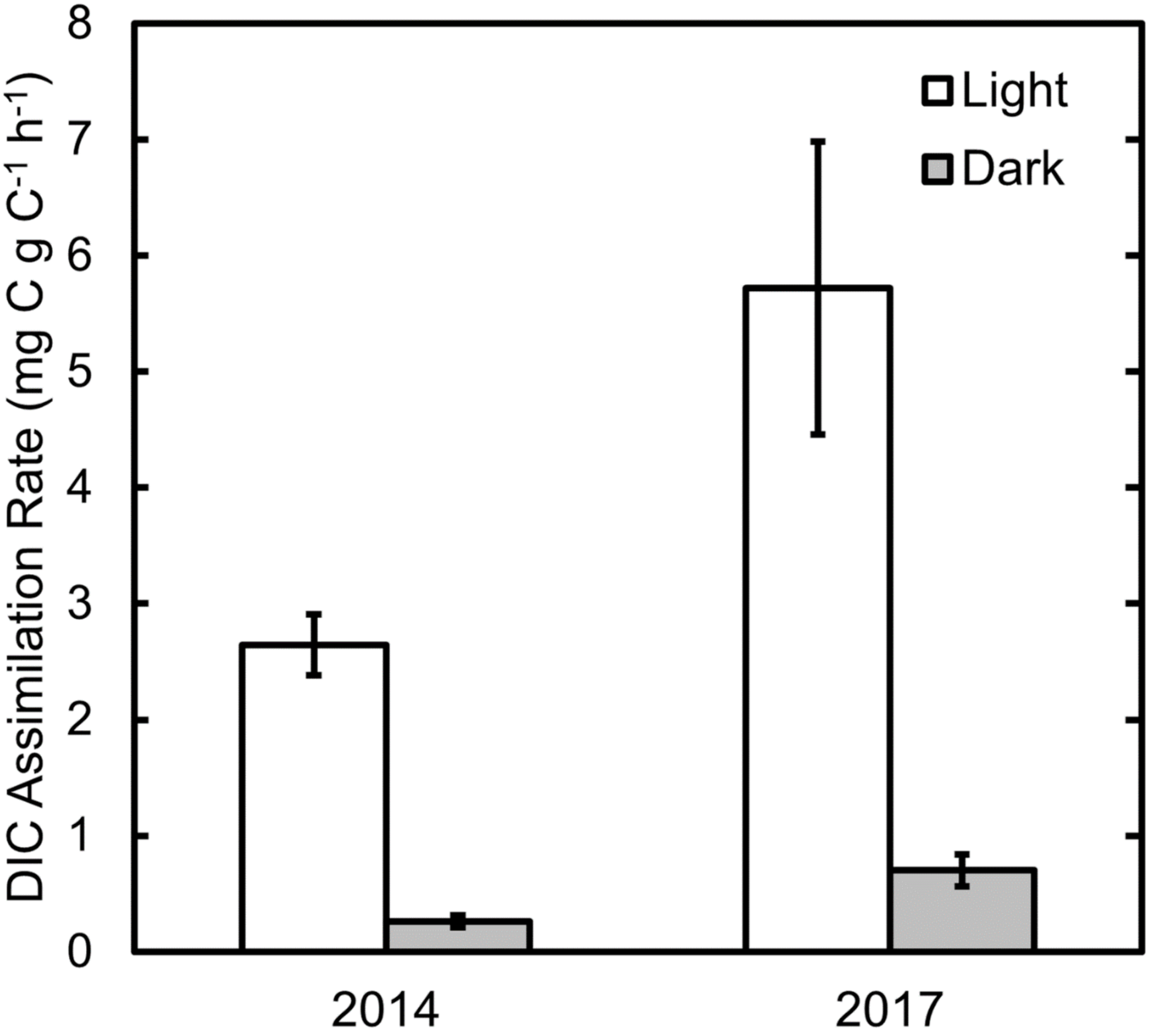

Figure S2. Rates of dissolved inorganic carbon (DIC) assimilation at the Amphitheater site in each year of this study measured in microcosms exposed to light (light) and microcosms excluded from light (dark). Microcosms were amended with 3-(3,4-dichlorophenyl)-1,1-dimethylurea (DCMU) in 2017; DCMU was not employed in 2014. Rates were calculated using DIC concentrations determined from filtered samples; see Methods and Table S3.

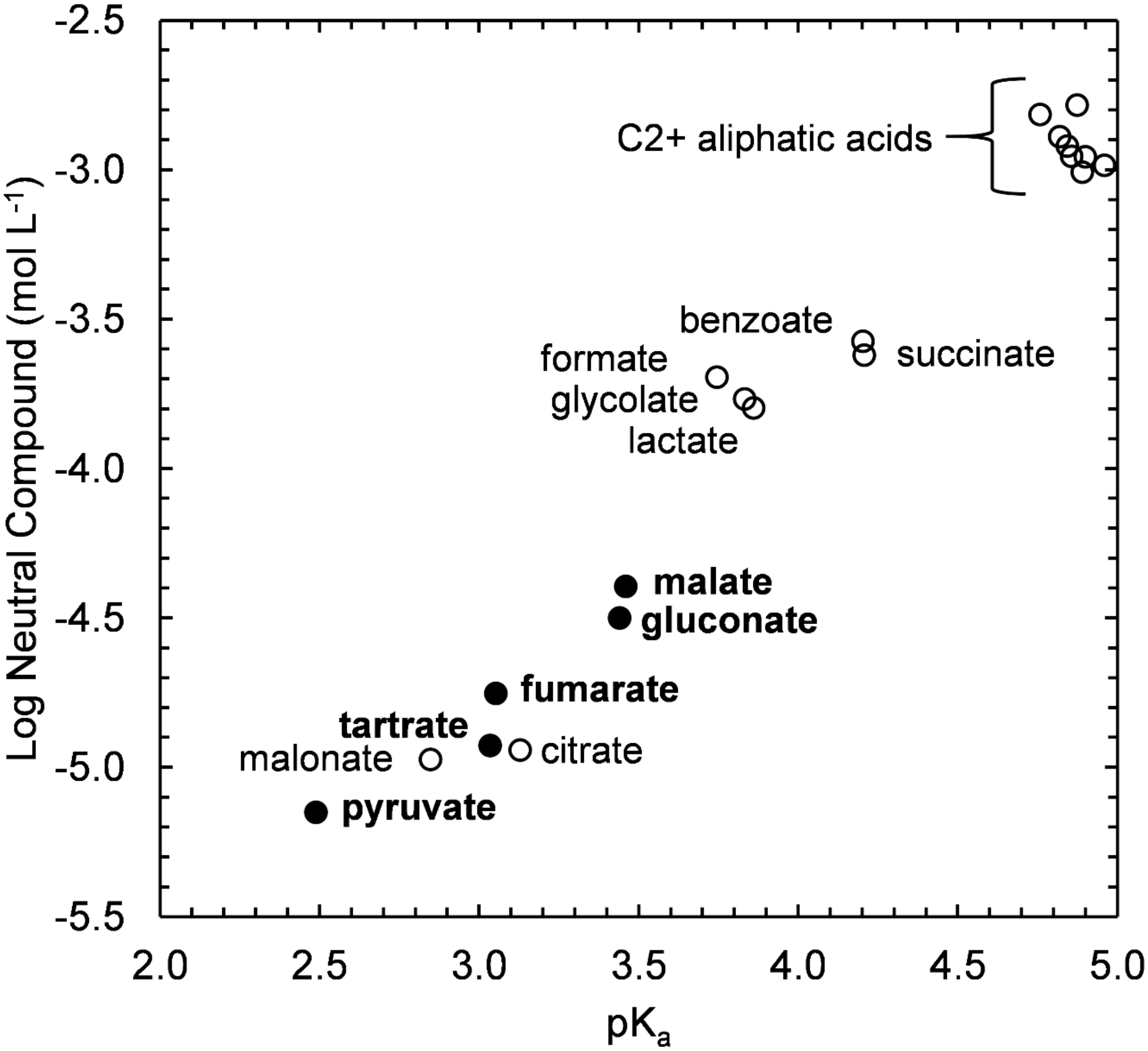

Figure S3. Calculated concentrations of the neutral (protonated) form of various carboxylic acids in growth experiments conducted by Pfennig (1974) plotted versus the pK_a of each carboxylic acid (first pK_a in the case of polyprotic acids). Compounds that supported growth of *R. globiformis* (filled circles) are indicated in bold font; compounds that inhibited growth (empty circles) are indicated in normal font. Succinate neither supported nor inhibited growth (succinate was indicated to be assimilated by Imhoff and Madigan (2021), however). Calculations are based on a culture medium pH of 5.6 and the addition of 1 g/L (0.1% w/w) of sodium salts of each carboxylate as indicated in Table S2. Assumptions were often required regarding which specific sodium salt was used; corresponding variations in the molar masses of the salt would not drastically affect the arrangement of the substrates in the figure. The other substrates tested by Pfennig (1974) not included in the figure are either 1) alcohols or sugars that have pK_a values >12 and thus are only negligibly ionized at all pH values of biological relevance or 2) amino acids (or amino acid salts) that are ionized at all pH values; in both cases the ionization state of the compound (*i.e.*, charged or uncharged) will not be different between the external cellular environment and the cytosol.

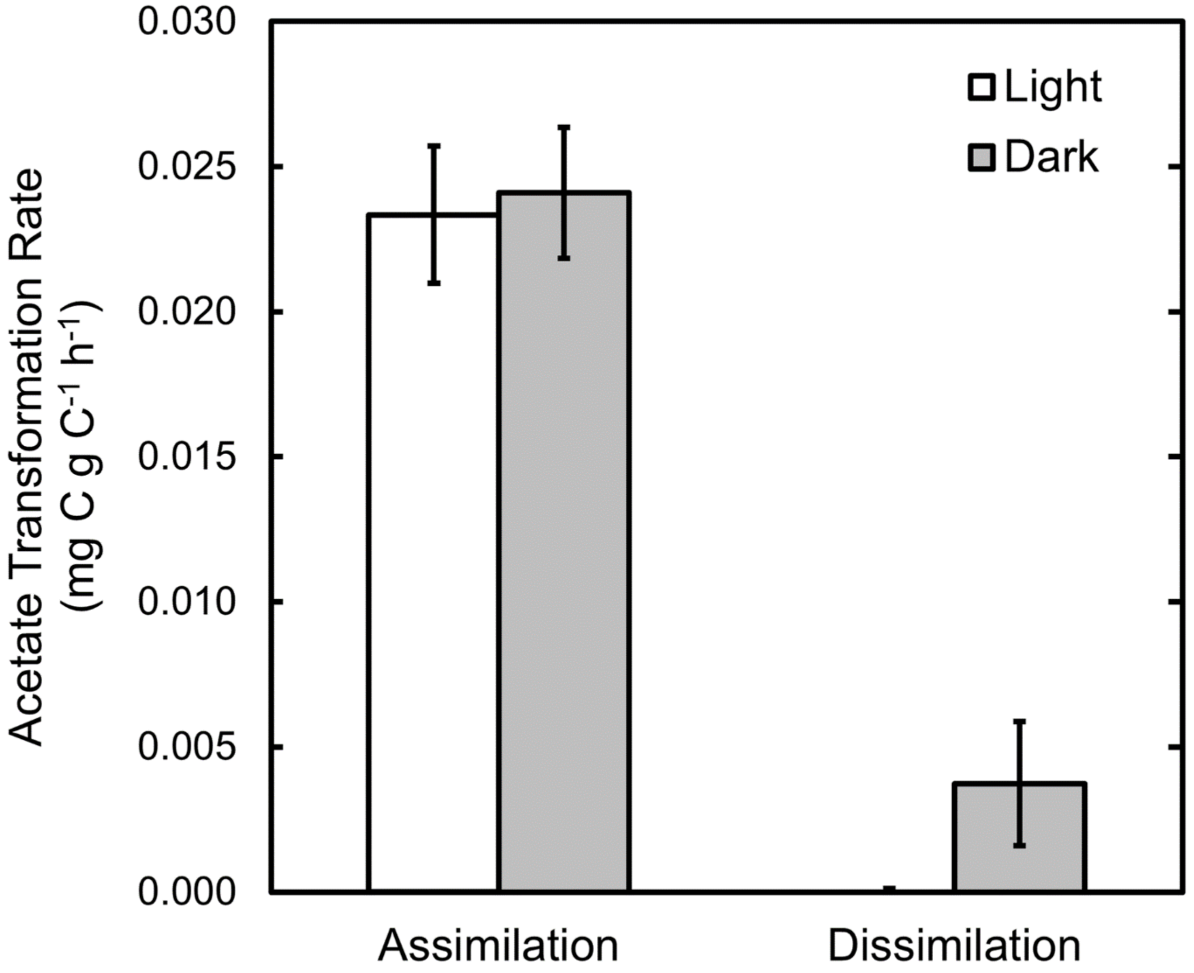

Figure S4. Rates of acetate assimilation (uptake into biomass) and dissimilation (mineralization, *i.e.* oxidation to inorganic carbon) at the Amphitheater site measured in microcosms exposed to light (light) and in microcosms excluded from light (dark). Rates were measured in 2017 (see Table S3). Notice that the rate of oxidation in the light is indistinguishable from zero, with the top of the error bar barely indicating a positive value.

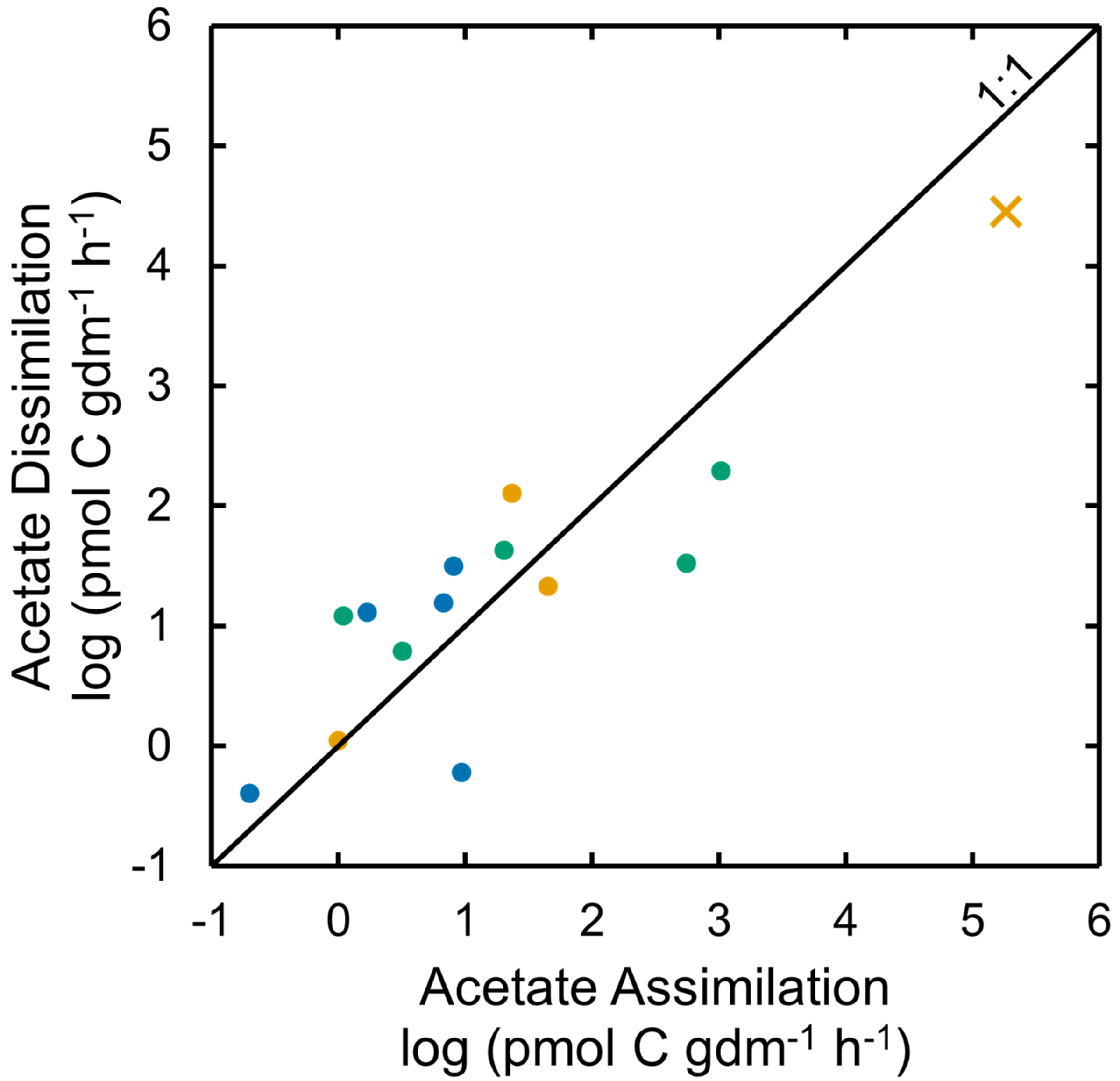

Figure S5. Rates of acetate dissimilation (mineralization, *i.e.* oxidation to inorganic carbon) versus rates of acetate assimilation (uptake into biomass) at the Amphitheater site in 2017 (cross; rate in Table S3 converted using mat carbon abundance reported in Table 1) and at various hot spring locations elsewhere in Yellowstone hosting chemotrophic (*i.e.*, T > 73°C) microbial communities (circles) reported by Urschel *et al.* (2015). Symbol color indicates the pH of the hot spring fluids at the sample location, where orange indicates pH < 4.0, blue indicates pH 4.0–6.9, and green indicates pH > 7.0, following Urschel *et al.* (2015). Rates were determined using microcosms excluded from light (wrapped in aluminum foil) and are normalized to the dry mass of sediments in the microcosms (grams dry mass, gdm). An equal rate line (1:1) is depicted for reference.

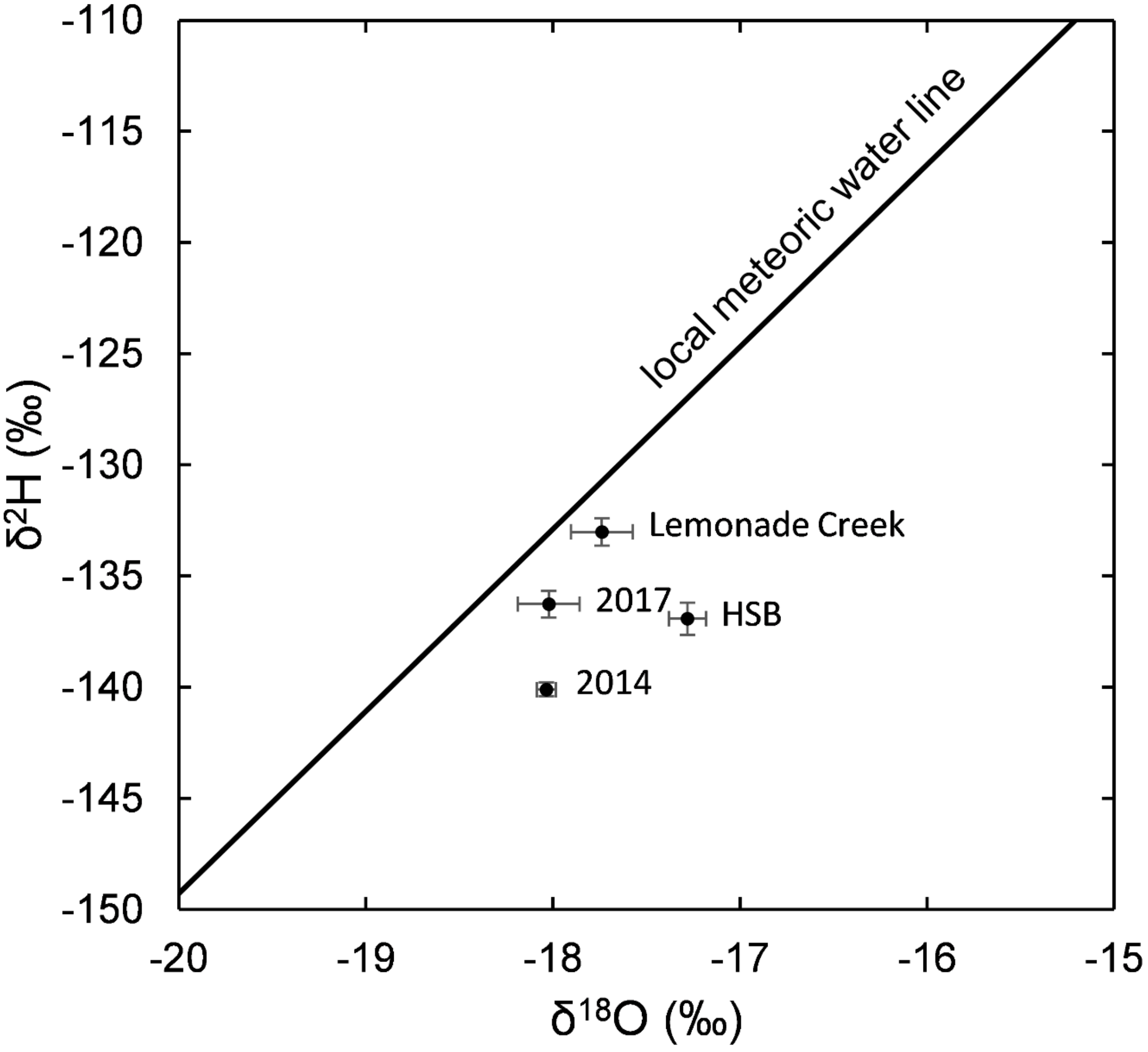

Figure S6. The isotopic composition of hydrogen (δ^2H) in H_2O versus the isotopic composition of oxygen ($\delta^{18}O$) in H_2O for natural waters analyzed in this study (Table 1; data in Table S5 not shown). Data for the Amphitheater site are labeled by year of sampling. Isotopic ratios are given relative to the Vienna Standard Mean Ocean Water (VSMOW) reference standard. Error bars represent one standard deviation based on five replicate analyses. The local meteoric water line is plotted for reference (Kharaka *et al.*, 2002; Holloway *et al.*, 2011).

Table S1. Summary of pigment results for mat biomass from the Amphitheater site.

Assignment	Retention Time (minutes)	Absorption Maxima (nm) [a]	%III/II [b]	Observed Molecular Ion (m/z)	Calculated $[M+H]^+$ (m/z)	Molecular Formula
fucoxanthin	6.6	(419), 446, (468)	0	659.4306	659.4306	C42H58O6
bacteriochlorophyll *a*	8.6	365, 606, 771		911.5529	911.5532	C55H74O6N4Mg
pheophorbide *a*	16.8	408, 506, 537, 609, 666		593.2762	593.2759	C35H36O5N4
cis-diatoxanthin [c]	20.8	437, 464	5	567.4201	567.4197	C40H54O2
bacteriopheophytin *a*	22.7	358, 528, 680, 748		889.5846	889.5838	C55H76O6N4
bacteriopheophytin *a'*	24.5	358, 528, 681, 749		889.5837	889.5838	C55H76O6N4
chlorophyll *a*	30.6	433, 478, 619, 665		893.5424	893.5426	C55H72O5N4Mg
pyropheophorbide *a*	37.6	409, 508. 539, 610, 667		535.2707	535.2704	C33H34O3N4
diatoxanthin [c]	44.6	(400), (425), 446, 474	44	567.4197	567.4197	C40H54O2
pheophytin *a* allomer	50.7	410, 510, 545, 623, 682		887.5683	887.5681	C55H74O6N4
pheophytin *a'*	54.2	407, 536, 609, 667		871.5732	871.5732	C55H74O5N4
pheophytin *a*	55.7	409, 505, 535, 609, 666		871.5747	871.5732	C55H74O5N4
β-carotene	56.6	452, 477	13	537.4458	537.4455	C40H56
cis-R.g. keto IV	59.3	(454), 481, 508	12	601.4621	601.4621	C41H60O3
cis-R.g. keto III	60.4	492, 521	6	629.4563	629.4570	C42H60O4
R.g. keto III	61.5	499, (525)	0	629.4571	629.4570	C42H60O4
R.g. keto IV	63.6	486, 513	3	601.4620	601.4621	C41H60O3
pyropheophytin *a*	64.1	667 [d]		813.5681	813.5677	C53H72O3N4
cis-R.g. keto II	65.4	(466), 492, 520	20	613.4621	613.4621	C42H60O3
cis-R.g. keto I	67.2	495, (524)	0	583.4516	583.4515	C41H58O2
dihydro-R.g. keto II	68.6	486, 512	14	615.4777	615.4772	C42H62O3
pheophytin *c*1	69.3	421		589.2449	589.2445	C35H32N4O5
cis-R.g. keto II	70.3	(464), 491, 521	8	613.4620	613.4621	C42H60O3
R.g. keto I	72.2	(460), 489, 513	1	583.4516	583.4515	C41H58O2
R.g. keto II	73.9	498, 527	13	613.4620	613.4621	C42H60O3
pheophytin *c*2	75.8	433, 498, 532, 574, 598		587.2291	587.2289	C35H30N4O5
lycopene	81.8	446, 473, 503	75	537.4464	537.4455	C40H56
3,4-didehydro-1,2-dihydrolycopene	83.7	457, 483, 515	70	ND [e]	537.4455	C40H56
3,4-didehydrolycopene	85.8	464, 495, 529	63	ND	535.4298	C40H54
3,4,3',4'-tetradehydrolycopene	87.6	(481), 509, 542	23	ND	533.4142	C40H52

[a]Maxima in parentheses indicate shoulders. [b]For carotenoid spectra, ratio of the peak heights of the highest-wavelength absorption maximum and the second-highest wavelength absorption maximum relative to the minimum absorbance between the two peaks, expressed as a percentage (Britton *et al.*, 2004). [c]Spectral data may also be consistent with the carotenoid monadoxanthin, a carotenoid associated with algae of the *Cryptophyceae* (Chapman, 1966; Pennington *et al.*, 1985; Britton *et al.*, 2004), however the prevalence of 18S rRNA gene sequences affiliated with *Bacillariophyta* (diatoms) and complete lack of sequences affiliated with *Cryptophyceae* weighs toward diatoxanthin. [d]Other absorption bands obscured by co-elution of R.g. keto IV. [e]Not detected.

Table S2. Speciation of carboxylates tested for growth of *R. globiformis* by Pfennig (1974) at a pH of 5.6 and 25°C[a] and 0.1% (w/w) concentration of each sodium salt.

Carboxylate	Growth[b]	pKa [c]	*M* of salt[d]	Total mM	Neutral mM	% Ionized
pyruvate	0-1	2.49	110.04	9.1	0.0070	99.9
malonate		2.847	166.04[e,f]	6.0	0.011	99.8
tartrate	1	3.036[g]	230.08[e,h]	4.3	0.012	99.7
fumarate	1	3.053	160.04[e]	6.2	0.018	99.7
citrate		3.128	294.10[h,i]	3.4	0.011	99.7
gluconate	3	3.44 [j]	218.14	4.6	0.031	99.3
malate	1	3.459	178.05[e]	5.6	0.040	99.3
formate		3.745	68.01	15	0.20	98.6
glycolate		3.832	98.03	10	0.17	98.3
lactate		3.860	112.06	8.9	0.16	98.2
benzoate		4.202	144.10	6.9	0.27	96.2
succinate	0	4.207	162.05[e]	6.2	0.24	96.1
acetate		4.757	82.03	12	1.5	87.4
butyrate		4.819	110.09	9.1	1.3	85.8
valerate		4.843[k]	124.11	8.1	1.2	85.1
caproate		4.857	138.14	7.2	1.1	84.7
propionate		4.874	96.06	10	1.6	84.2
caprylate		4.89 [k]	166.19	6.0	0.98	83.7
cyclohexanecarboxylate		4.899[k]	150.15	6.7	1.1	83.4
pelargonate		4.96 [l]	180.20	5.5	1.0	81.4

[a]Incubation temperature was 28°C (Pfennig, 1974); the effect of this temperature difference on the pK_a values is negligible. [b]Growth designations per Pfennig (1974): 0, optical density (OD) at 650 nm same as control; 1, OD up to 0.1; 3, OD 0.6–0.9 or higher; blank entries indicate the substrate completely inhibited growth. [c]pK_a values from Smith and Martell (1989) and correspond to infinite dilution unless otherwise indicated; the first pK_a is given in the case of polyprotic acids. [d]Molar mass of the sodium carboxylate salt assumed to have been employed (anhydrous unless otherwise indicated) in grams/mole. [e]Dibasic. [f]Monohydrate. [g]D-Tartaric acid. [h]Dihydrate. [i]Tribasic. [j]0.1 ionic strength. [k]Martell and Smith, 1977. [l]Rumble, 2021.

Table S3. Rates of dissolved inorganic carbon and acetate transformation at the Amphitheater site.

Year	Substrate	Transformation	DIC basis	Light mg C g C^{-1} h^{-1}	Light uncertainty	Dark mg C g C^{-1} h^{-1}	Dark uncertainty
2017	DIC	assimilation	unfiltered	7.8	1.7	0.96	0.19
2017	DIC	assimilation	filtered	5.7	1.3	0.70	0.14
2014	DIC	assimilation	filtered	2.6	0.26	0.33	0.049
2017	Acetate	assimilation	N/A	0.023	0.0024	0.024	0.0023
2017	Acetate	dissimilation	N/A	-0.00010	0.00020	0.0037	0.0021

Table S4. Rates of dissolved inorganic carbon assimilation for other sites.

Site	Light −DCMU mg C g C^{-1} h^{-1}	Light −DCMU uncertainty	Light +DCMU mg C g C^{-1} h^{-1}	Light +DCMU uncertainty	Dark mg C g C^{-1} h^{-1}	Dark uncertainty
SSA1 [a]	6.1	1.8	0.097	0.0072	0.060	0.015
SSA2 [a]	6.7	3.9	0.020	0.019	0.060	0.015
RS6	1.2	0.33	0.060	0.045	0.0084	0.0058
Dragon	> 1.3 [b]	0.082	0.028	0.0026	0.015	0.0024

[a]Data from Hamilton *et al.* (2019). [b]Activities in microcosms exceeded the range of the scintillation counter; rate is a minimum value.

Table S5. Physicochemical data for additional sites

	RS6	Dragon Spring Outflow
Sample ID (YYMMDD)	140805TM	170725TJ
Easting	0515136	0552889
Northing	4928588	4953212
Elevation, m	2270	2280
Temperature, °C	49.7	49.8
Specific conductance, μS/cm	580	2129
pH (field / calculated)	2.94 / 2.90	3.01 / 3.08
Sulfide, μM	0.053	0.056
Dissolved oxygen, μM	31	110
Silica, mM	1.7	6.2
Fluoride, mM	0.025	0.16
Chloride, mM	0.012	12
Bromide, μM	0.030	17
Sulfate, mM	1.2	1.5
Nitrate, μM	0.55	0.087
Lithium, μM	1.4	510
Sodium, mM	0.32	12
Potassium, mM	0.21	1.3
Magnesium, mM	0.018	0.0096
Calcium, mM	0.044	0.12
Ammonium, μM	230	79
Ferrous iron, μM	6.1	35
Δ^2H-H_2O, ‰ vs. VSMOW	−115.1 ± 0.1	−139.0 ± 0.7
$\delta^{18}O$-H_2O, ‰ vs. VSMOW	−6.51 ± 0.01	−14.5 ± 0.2
DIC, mM	0.38	0.30
$\delta^{13}C$-DIC, ‰ vs. VPDB	0.7 ± 0.8	−6.1 ± 0.4
DOC, mM	0.044	0.073
$\delta^{13}C$-DOC, ‰ vs. VPDB	−25.3 ± 0.2	−24.7 ± 0.2
Mat C, wt. % (dry mass basis)	2.56 ± 0.02	7.94 ± 0.07
$\delta^{13}C$-Mat, ‰ vs. VPDB	−19.1 ± 0.1	−11.85 ± 0.09
Mat N, wt. % (dry mass basis)	0.332 ± 0.001	1.404 ± 0.003
$\delta^{15}N$-Mat, ‰ vs. air	−5.9 ± 0.2	−7.04 ± 0.08
Sediment C:N, mol:mol	9.01 ± 0.09	6.60 ± 0.06
$\Delta^{13}C$, ‰	−19.8 ± 0.8	−5.8 ± 0.4
Charge imbalance, %	−6.6	1.3

References

Britton, G., Liaaen-Jensen, S., and Pfander, H. eds. (2004) Carotenoids Handbook, Springer Basel AG.

Chapman, D.J. (1966) Three new carotenoids isolated from algae. *Phytochemistry* **5**: 1331–1333.

Hamilton, T.L., Bennett, A.C., Murugapiran, S.K., and Havig, R. (2019) Anoxygenic phototrophs span geochemical gradients and diverse morpologies in terrestrial geothermal springs. *mSystems* **4**: e00498-19.

Holloway, J.A.M., Nordstrom, D.K., Böhlke, J.K., McCleskey, R.B., and Ball, J.W. (2011) Ammonium in thermal waters of Yellowstone National Park: Processes affecting speciation and isotope fractionation. *Geochim Cosmochim Acta* **75**: 4611–4636.

Imhoff, J.F. and Madigan, M.T. (2021) Rhodopila. In, *Bergey's Manual of Systematics of Bacteria and Archaea*. Wiley.

Kharaka, Y.K., Thorsden, J.J., and White, L.D. (2002) Isotope and chemical compositions of meteoric and thermal waters and snow from the greater Yellowstone National Park region. *Open File Rep* **02–194**: 1–76.

Martell, A.E. and Smith, R.M. (1977) Critical Stability Constants. Volume 3: Other Organic Ligands, New York: Plenum Press.

Pennington, F.C., Haxo, F.T., Borch, G., and Liaaen-Jensen, S. (1985) Carotenoids of cryptophyceae. *Biochem Syst Ecol* **13**: 215–219.

Pfennig, N. (1974) Rhodopseudomonas globiformis, sp. n., a new species of the Rhodospirillaceae. *Arch Microbiol* **100**: 197–206.

Rumble, J.R. ed. (2021) CRC Handbook of Chemistry and Physics, 102nd ed. Boca Raton, FL: CRC Press/Taylor & Francis.

Smith, R.M. and Martell, A.E. (1989) Critical Stability Constants. Volume 6: Second Supplement, New York: Plenum Press.

Urschel, M.R., Kubo, M.D., Hoehler, T.M., Peters, J.W., and Boyd, E.S. (2015) Carbon source preference in chemosynthetic hot spring communities. *Appl Environ Microbiol* **81**: 3834–3847.

Appendix

Photographs of the Amphitheater spring

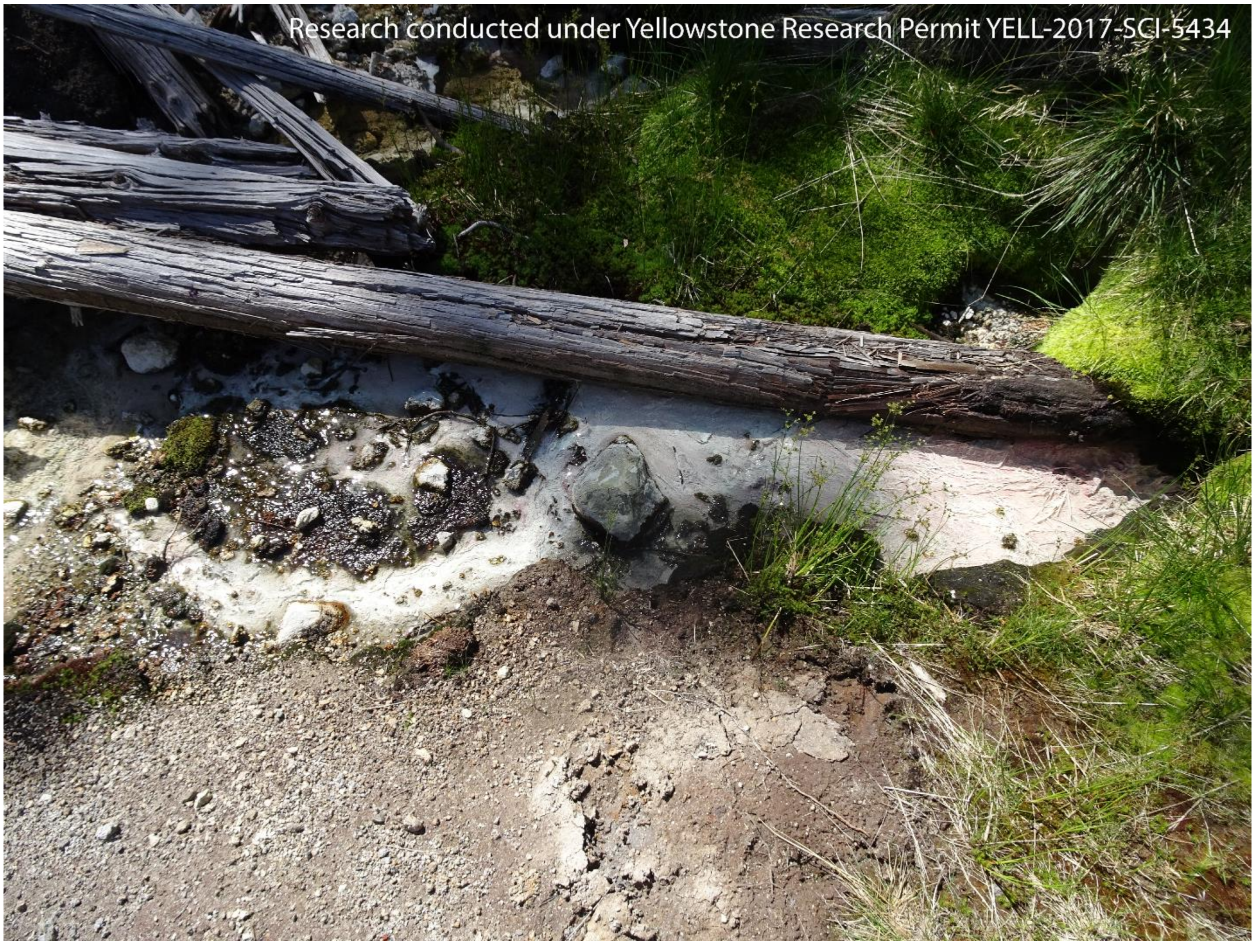

7/22/2017 (KMF photo)

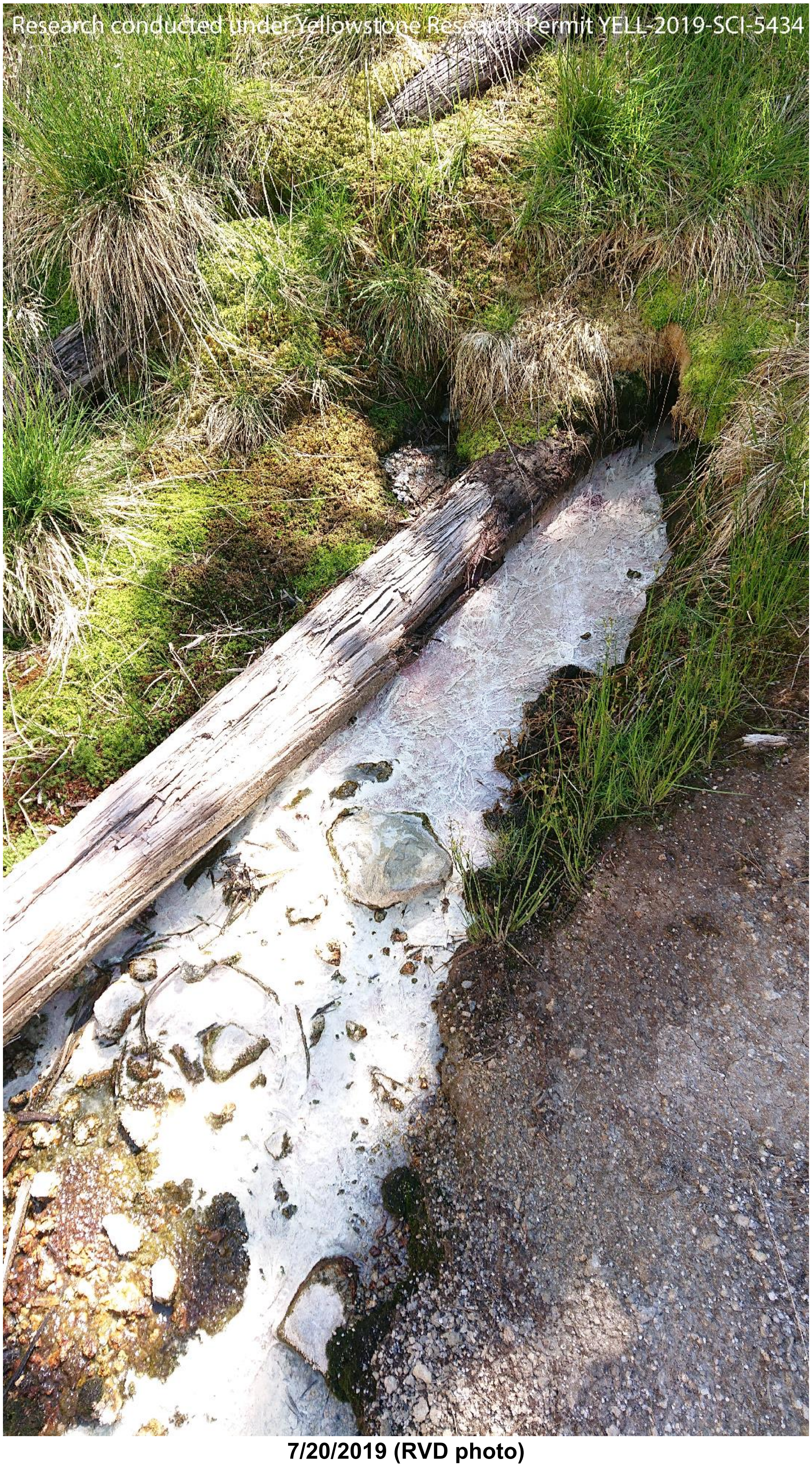

7/20/2019 (RVD photo)

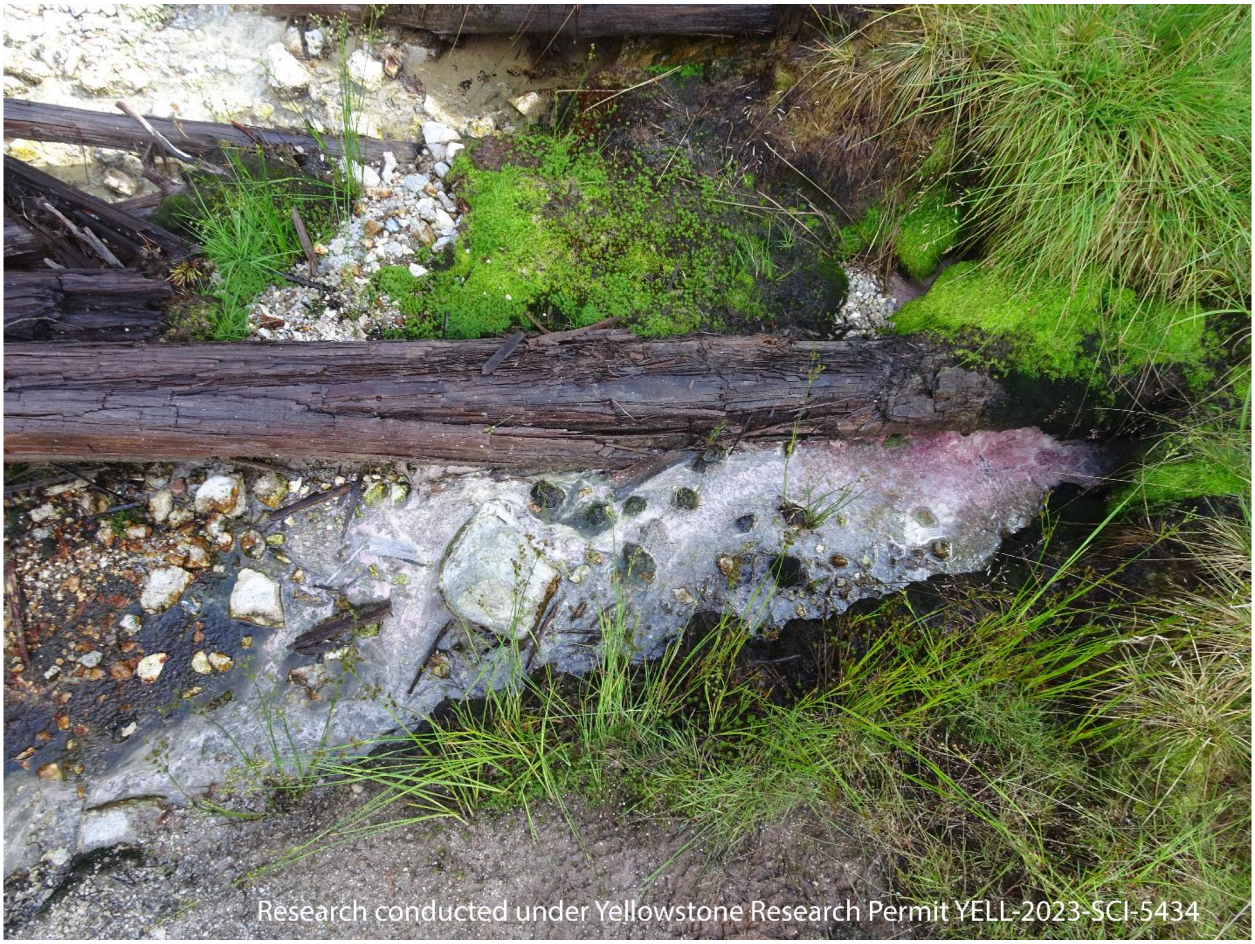

8/5/2023 (KMF photo)

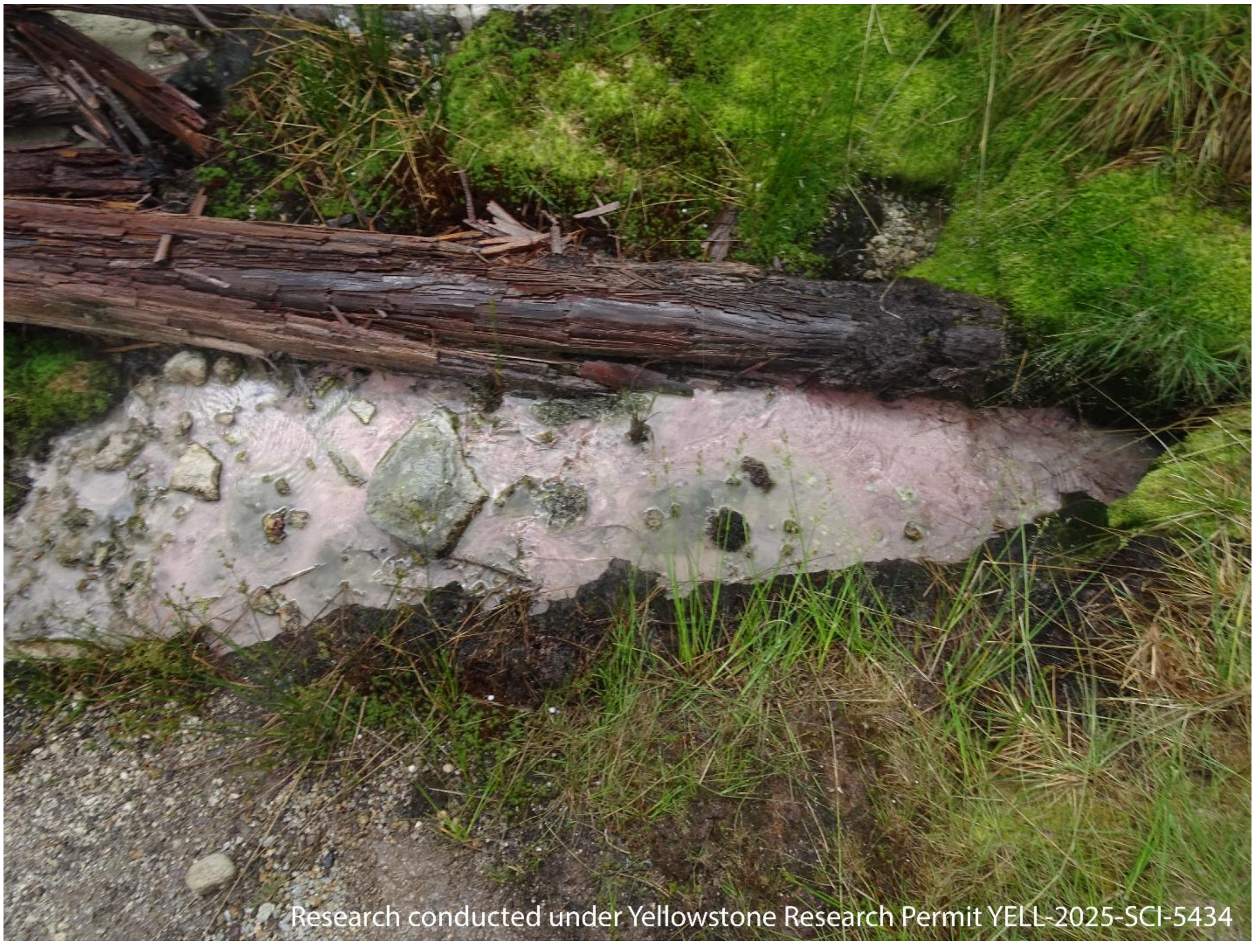

7/18/2025 (KMF photo)
